# Immune Cell Infiltration and Kynurenine Pathway Activation Define Early Injury and Progression in Diabetic Nephropathy

**DOI:** 10.7150/ijbs.122164

**Published:** 2026-01-08

**Authors:** Yi-Chun Tsai, Chia-Yen Dai, Ming-Ju Tsai, Shang-Jyh Hwang, Ping-Shaou Yu, Wei-Wen Hung, Sheng-Feng Pan, Ling-Yu Wu, Pei-Hsun Tsai, Hung-Pei Tsai, Wei-An Chang, Ya-Ling Hsu

**Affiliations:** 1 School of Medicine, College of Medicine, Kaohsiung Medical University, Kaohsiung 807, Taiwan.; 2 Division of Nephrology, Kaohsiung Medical University Hospital, Kaohsiung Medical University, Kaohsiung, Taiwan.; 3 Department of Internal Medicine, Kaohsiung Municipal Cijin Hospital, Kaohsiung, Taiwan.; 4 Division of Hepato/Billiary/Pancreatic, Kaohsiung Medical University Hospital, Kaohsiung Medical University, Kaohsiung 807, Taiwan.; 5 Division of Pulmonary and Critical Care Medicine, Department of Internal Medicine, Kaohsiung Medical University Hospital, Kaohsiung Medical University, Kaohsiung 807, Taiwan.; 6 Division of Endocrinology and Metabolism, Department of Internal Medicine, Kaohsiung Medical University Hospital, Kaohsiung Medical University, Kaohsiung, Taiwan.; 7 Graduate Institute of Medicine, College of Medicine, Kaohsiung Medical University, Kaohsiung 807, Taiwan.; 8 Division of Neurosurgery, Department of Surgery, Kaohsiung Medical University Hospital, No.100, Tzyou 1 st Road Kaohsiung 807378, Taiwan.; 9 Drug Development and Value Creation Research Center, Kaohsiung Medical University, Kaohsiung 807, Taiwan.

**Keywords:** diabetic nephropathy, MDSC, kynureninase, kynurenine pathway

## Abstract

Diabetic nephropathy (DN), a major complication of diabetes, is the leading cause of end-stage kidney disease; however, a comprehensive understanding of the dynamic immune-inflammatory changes during DN progression remains limited. We integrated single-cell RNA sequencing, in vivo diabetic models, and clinical samples from type 2 diabetes (T2D) patients to investigate cellular and molecular alterations across different stages of DN. Our results revealed a significant increase in immune cell infiltration in early DN in two mouse models. Notably, CCL4⁺ myeloid-derived suppressor cells (MDSCs) with a pro-inflammatory phenotype emerged as the predominant infiltrating immune population, with S100A9 highly expressed in these cells, serving as early molecular indicators. Compared to healthy individuals, T2D patients exhibited elevated levels of circulating MDSCs. Long non-coding RNA MALAT1 was identified as a key factor in maintaining MDSC function. In late DN, elevated expression of CD9 and TREM2 in kidney macrophages suggested a role for lipid-associated macrophages in DN progression. Concurrently, endothelial cell reprogramming characterized by GPX3 and SPP1 expression was observed during DN advancement. Furthermore, kynureninase, a key enzyme in the kynurenine pathway (KP), was upregulated in proximal tubule injury during early DN. The metabolites of KP including kynurenine, 3-hydroxykynurenine, and quinolinic acid were associated with enhanced induction of MDSCs in mice, as well as with adverse renal outcomes in T2D patients. Collectively, this study delineates the dynamic immune-inflammatory landscape of DN, uncovers key molecular players across disease stages, and provides novel insights into potential diagnostic markers and therapeutic targets.

## Introduction

Diabetic nephropathy (DN) is a major complication of diabetes that significantly impacts kidney function and is the leading cause of chronic kidney disease worldwide 1, 2. An estimated 415 million people worldwide were living with diabetes, contributing to 5 million deaths and around US$673 billion in global healthcare costs in 2015 3, 4. The prevalence of diabetes is projected to rise to 642 million people by 2040, with 30-40% expected to develop DN 5. While recent therapies like SGLT2 (sodium-glucose cotransporter 2) inhibitors have shown promise in managing DN, there is still an urgent need for kidney-targeted treatments that can effectively halt or reverse disease progression. Therefore, the complex relationship between obesity, type 2 diabetes (T2D), and kidney function progression remains not fully understood.

The development of DN is multifaceted, involving a wide range of interconnected biological pathways 6. In recent years, growing attention has been given to the role of chronic inflammation and immune cells in the development and progression of DN 7. Studies have consistently shown elevated levels of pro-inflammatory cytokines in the blood, urine, and kidney tissues of individuals with diabetes 8, 9. Immune cell infiltration is a common feature observed in kidney biopsy samples across all stages of DN, affecting both the glomeruli and the interstitial areas 10, 11. Accumulating evidence indicates that tubulointerstitial inflammation, with a particular emphasis on the involvement of proximal tubular epithelial cells (PTECs), plays a pivotal role in the pathogenesis of DN 12, 13. Elevated expression levels of inflammatory cytokines, chemokines, adhesion molecules, and growth factors have been consistently observed in PTECs of kidney specimens from individuals or mouse with DN 14, 15. However, unraveling the intricate relationship between immune cells and renal cells remains unclear, especially regarding how immune cells are recruited and infiltrate the kidney, as well as how they interact with resident kidney cells. The persistent inflammatory environment is sustained by a complex network of signaling pathways and cytokines, further complicating efforts to fully understand the mechanisms involved.

To address these gaps in knowledge, this study employs single-cell transcriptomic analysis to dissect the cellular landscape of the diabetic kidney at high resolution. By characterizing cell-type-specific transcriptional alterations, particularly in immune cells and their surrounding microenvironment, we aim to uncover novel insights into the cellular interactions and regulatory networks that contribute to DN pathogenesis. This approach provides a comprehensive framework for understanding the heterogeneity of kidney inflammation in diabetes and may ultimately inform the development of more precise and effective therapeutic strategies.

## Materials and Methods

### Animal model

Two distinct murine models were employed in our previous studies to investigate diabetes using single-cell RNA sequencing analysis and following experiments 16. The first model included male C57BL/KsJ-db/m mice as controls and C57BL/KsJ-db/db mice as diabetic subjects, with kidney tissues collected at 14 weeks (representing the early-stage), 22 weeks (mid-stage), and 33 weeks (late-stage). The second model utilized C57BL/6 male mice, which were placed on a high-fat diet (HFD, 60% fat content) for 12 weeks starting at 8 weeks of age, followed by an intraperitoneal injection of streptozotocin (STZ) at 100 mg/kg to induce diabetes, or on chow diet as control group. These mice were sacrificed at 28 weeks of age, and kidney tissues were formalin-fixed and paraffin-embedded (FFPE) for histological analysis. Blood glucose levels were routinely monitored via tail vein sampling 16. All animals were obtained from the National Laboratory Animal Center, and all procedures were approved by the Institutional Animal Care and Use Committee (IACUC) of Kaohsiung Medical University (KMU) (approval no. 110131).

### Single-cell RNA sequencing (scRNA-seq) analysis

We used db/m and db/db mice at 14 and 33 weeks, C57BL/6 and HFD/STZ mice for scRNA-seq (n = 3 at each group). FFPE kidney blocks obtained from control and HFD/STZ-treated mice were sectioned into 50 μm curls. Tissue dissociation was performed using the Miltenyi Biotec FFPE Tissue Dissociation Kit (Cat. No. 130-118-052) in accordance with the CG000233 protocol provided by 10X Genomics (Pleasanton, CA, USA). The resulting cell suspensions were processed using the Chromium X system, following the instructions outlined in the Chromium Fixed RNA Profiling for Multiplexed Samples guide (CG000527). cDNA libraries were generated and sequenced on an Illumina NovaSeq 6000 platform (San Diego, CA, USA) using paired-end dual-indexing. The single-cell RNA-seq data from the kidneys of db/m and db/db mice were processed as described in our previous study 17. Sequencing data from all libraries were demultiplexed using Illumina's bcl2fastq software, and downstream analysis was carried out with Cell Ranger version 7.1.0 (10X Genomics). Specifically, the Cell Ranger "count" pipeline was applied to each microbead well using the mm10-2020A reference genome to generate gene-barcode matrices and other relevant outputs.

Single-cell count matrices were preprocessed with Scanpy (version 1.10.2), following Single-Cell Best Practices. For the HFD/STZ diabetic model, outliers were removed using thresholds at ±5 times the median absolute deviation (MAD) on per-cell QC metrics; for the genetic db/db model, asymmetric thresholds were applied, excluding cells above +4 times the MAD or below -5 times the MAD. In both datasets, cells with > 30% mitochondrial gene content (mouse mt-genes) were excluded. Ambient RNA was corrected per library with SoupX (version 1.6.2) prior to normalization and integration. Doublets were then identified and removed using the SOLO model in scvi-tools (version 1.1.6). Highly variable genes (n = 2000) were selected using the Seurat v3 method as implemented in Scanpy, and a latent space was learned with scvi-tools. A k-nearest-neighbor graph built on the scVI latent space was used to compute Leiden clusters and Uniform Manifold Approximation and Projection (UMAP) embeddings. Finally, the gene matrix was converted with loupeR (version 1.1.4) into a format compatible with Loupe Browser (version 8.0) for subcluster exploration and differential gene expression (DEG) analysis.

### Bioinformatics

The cell subpopulations were identified using characteristic marker genes, guided by references from the original study as well as annotations provided in the TISCH2 (http://tisch.comp-genomics.org/gallery/) database and CellMarker 2.0 (http://117.50.127.228/CellMarker/CellMarker_help.html). Genes were considered as signature markers if they were detected in more than 50% of the cells within at least one specific cell population and showed a log2 fold change greater than 1, indicating at least a two-fold upregulation. To explore the biological significance of these genes, pathway enrichment analysis was conducted using the Kyoto Encyclopedia of Genes and Genomes (KEGG) database. Additionally, to investigate genes that may be involved in cellular transitions or differentiation processes, trajectory inference was performed using Monocle 2 (v2.22).

### The preparation of macrophage and myeloid-derived suppressor cells (MDSCs)

After harvesting kidneys from 33-week-old db/m and db/db mice, the tissues were enzymatically digestion by Multi Tissue Dissociation Kit 2 ((130-110-203; Miltenyi Biotec) into single-cell suspensions. Macrophages were then isolated by first enriching CD11b⁺ cells, followed by purification using anti-F4/80 microbeads. Splenic MDSCs of healthy mice were isolated using a mouse MDSC isolation kit (Miltenyi Biotec) following the manufacturer's protocol. To mimic diabetic stress conditions, splenic immune cells were subjected to various treatment conditions, including normal glucose (NG, 5.5 mM), high glucose (HG, 25 mM), and palmitic acid (PA, 50 µM). In addition, a combined treatment of HG and PA was applied for 48 hours. Following incubation, cells were collected for further analysis using specialized downstream assays.

### Quantitative PCR (qPCR)

To assess mRNA expression, cells were collected and total RNA was isolated using the TRIzol reagent. 50 ng of RNA was reverse transcribed into cDNA RT Reagent Kit (Cat RR037A, TaKaRa), following the manufacturer's protocol. Quantitative PCR was conducted in three technical replicates per biological replicate, using 1 µL of cDNA per reaction. 18S rRNA served as the internal control and the primer sequences were listed in Table S1 on a QuantStudio 3 machine (Applied Biosystems). 18S rRNA was used as the internal control gene, and relative fold changes in gene expression were calculated using the 2^-ΔΔ^Ct method.

### Immunofluorescence (IF) and immunohistochemistry (IHC) validation

Optimal cutting temperature or paraffin-embedded kidney tissues (from both human and mouse samples) were stained using primary antibodies targeting kidney injury molecule 1 (KIM-1, Cat ab78494, Abcam), CD45 (Cat CD45(AKYP0005)-BX007-ALEXA FLUOR™ 488, Akoya biosciences), kynureninase (Cat 11796-1-AP, Proteintech for human; Cat MAB7389, R&D system for mouse), CD31 (Cat (AKYP0002)-BX002, Akoya biosciences), glutathione peroxidase 3 (GPX3, Cat 13947-1-AP, ProteinTech), and secreted phosphoprotein 1 (SPP1, Cat 88742, Cell Signaling Technologies). For IHC stain, sections were incubated with the primary antibodies followed by a secondary antibody treatment. In the case of IF, samples were incubated with a fluorescently labeled secondary antibody mix, along with an anti-fade mounting medium containing DAPI. The stained tissues were then examined using a Zeiss LSM700 Confocal microscope (Zeiss, MJena, Germany).

### MDSC differentiation from mouse bone marrow cells

Bone marrow cells were isolated from the femurs and tibias of 10-week-old C57BL/6 mice under sterile conditions. After red blood cell lysis, the cells were cultured in RPMI 1640 medium supplemented with 10% fetal bovine serum (FBS). Cells were seeded at a density of 1 × 10⁶ cells/mL and treated with 3-hydroxyanthranilic acid (3-HAA, 50 μM), quinolinic acid (QA, 50 μM), or anthranilic acid (AA, 50 μM). Following treatment, cells were harvested and stained with fluorochrome-conjugated antibodies against anti-mouse CD45 (APC), CD11b (BV421), LY6C (lymphocyte antigen 6 family member C, FITC), and LY6G (PE) (all from BD Biosciences). Flow cytometry was performed using a BD FACSLyric, and data were analyzed using FlowJo software to assess the proportion of CD45+ CD11b⁺Ly6C⁺Ly6G⁺ MDSCs.

### Enzyme-linked immunosorbent assay (ELISA) and Luminex system

The serum of mice and the cell supernatants obtained from in situ experiments were tested for chemokine (C-X-C motif) ligand 1 (CXCL1), CXCL2, Interleukin-1 beta (IL-1β), S100 calcium-binding protein A8 (S100A8), and S100A9 using a Luminex microbead-based multiplex assay (R&D Systems). The concentrations of SPP1 lipocalin 2 (LCN2), and Serglycin (SRGN) were measured using Mouse & Rat Osteopontin (Cat MOST00, R&D system), mouse Lipocalin-2/NGAL (Cat MLCN20, R&D system) and mouse SRGN (Cat EK247187, AFG Bioscience) ELISA kits.

### GPX3 overexpression and metastasis associated lung adenocarcinoma transcript 1 (MALAT1) knockdown

Mouse bone marrow cells were then subjected to electric field pulse treatments (40 nM control (Cat D-001320-10-20, Dharmacon) or MALAT1 siRNA (Cat R-174310-00-0020, Dharmacon)) using a BTX ECM 2001 Electroporation System (Harvard Apparatus). To achieve high transfection efficiencies and cell viabilities, the optimal parameters for electric pulse sequences were a 200 V/cm field strength and a 70 msec duration. S100A9 and LCN2 mRNA levels were measured by qPCR. Glomerular endothelial cells (GECs, Cat 4000, Sciencell) were cultured in Endothelial Cell Medium (Cat 1001, Sciencell) plus 5% FBS, according to the manufacturer's suggestions. GECs were transfected with control cDNA (Cat SKU PS100010) or GPX3 cDNA (Cat SKU MG222219, Origene) for 48 h, the cell supernatant was collected and the SPP1 level was measured by ELISA.

### Human study subjects

Patients with T2D attending the outpatient departments of Kaohsiung Medical University Hospital (KMUH) were invited to participate in this prospective study from October 2016 to December 2023. T2D was diagnosed based on medical history, American Diabetes Association-defined blood glucose levels, or prescriptions for anti-diabetic medications. All enrolled patients received a diabetes education program and adhered to the recommended dietary guidelines for individuals with diabetes. Estimated glomerular filtration rate (eGFR) was determined using the 2009 Chronic Kidney Disease Epidemiology Collaboration (CKD-EPI) formula 18. The exclusion criteria were eGFR below 30 ml/min/1.73 m², acute illness, and the use of antimicrobial or probiotic agents in the month prior to enrollment. In addition, we collected kidney sections obtained from three patients with DN scheduled for biopsies at KMUH from November 2016 to August 2020 and three patients with upper tract urothelial carcinoma (UTUC) who underwent nephrectomy at Kaohsiung Municipal Ta-Tung Hospital from October 2018 to August 2023. Approval for this study was granted by the Institutional Review Board of KMUH (KMUHIRB-20130089, KMUHIRB-G(II)-20150044, KMUHIRB-G(I)-20160017, KMUHIRB-G(II)-20160021, KMUHIRB-G(II)-20190036). All enrolled patients provided written informed consent, and the study was conducted following the principles of the Declaration of Helsinki.

The information of clinical characteristics including demographics, smoking and alcohol history, and clinical history was collected from interviews and medical records of the patients at enrollment. Hypertension was diagnosed based on medical history or prescriptions for antihypertensive medications. Body mass index was calculated as body weight/height^2^. Data on medication usage were recorded from medical records before and after enrollment, including anti-diabetic drugs, anti-hypertensive drugs, insulin, and statins. Twelve-hour fasting blood and urine samples were obtained for biochemical analysis. The compensated Jaffé (kinetic alkaline picrate) method was used to measure serum creatinine, which was conducted using an autoanalyzer (Roche/Integra 400, Roche Diagnostics) and isotope-dilution mass spectrometry calibrator (Vickery, Stevens, Dalton, van Lente, & Lamb, 2006). The urinary albumin/creatinine ratio (UACR) was used to express the amount of protein in urine.

### Flow cytometry analysis of MDSCs in human peripheral blood mononuclear cells (PBMC)

Peripheral blood samples were collected from patients with T2D (n=5) and age and sex-matched healthy controls (n=6), following informed consent and institutional ethical approval (KMUHIRB-E(II)-20210076, KMUHIRB-G(I)-20180032, KMUHIRB-G(II)-20160024). PBMCs were isolated by density gradient centrifugation using Ficoll-Paque PLUS (GE Healthcare). After isolation, PBMCs were washed and resuspended in staining buffer, followed by incubation with fluorochrome-conjugated antibodies against lineage markers (CD3, CD14, CD16, CD19, CD20, CD56), CD45, CD11b, and CD33 (all from BD Biosciences or BioLegend). Human MDSCs were defined as lineage-CD45⁺CD11b⁺CD33⁺ cells. Flow cytometry was performed using a The BD FACSLyric™, and data were analyzed with FlowJo software.

### Ultra-high performance liquid chromatography-tandem mass spectrometry (UHPLC-MS/MS)

Serum sample was added with methanol containing internal standard. The samples were placed at 4 °C for 30 mins, followed by centrifugation at 5000 rpm at 4 ℃ for 5 min. All the sample solutions were filtered through a 0.2 μm PVDF membrane filter and then analyzed by UHPLC-MS/MS that was performed on a SHIMADZU LC-20ADxr system coupled with a SCIEX QTrap® 5500 mass spectrometry system. Separation was achieved on a HSS-T3 column (2.1 mm × 100 mm, 1.7 μm) with a mobile phase consisting of 0.1% formic acid in water (solvent A) and 0.1% formic acid acetate in ACN (solvent B) at a flow rate of 0.35 mL min-1. To prepare the calibration curve, the mixed standard solution contained tryptophan, kynurenine, QA, 3-Hydroxykynurenine (3-HK), kynurenic acid (KA), and xanthurenic acid (XA) was serially diluted, ranging from 1 to 2000 ng/mL with 50% methanol and followed the same sample preparation protocol mentioned above. Data obtained from the SCIEX QTrap® 5500 mass spectrometry system were converted into comma-separated values (csv) files and processed with SCIEX OS software.

### Clinical outcomes of the study subjects

The patients were followed until kidney outcome was recorded, death, last contact, or the end of the follow-up period (December 2024). The patients visited an outpatient clinic every three months, when their clinical status and eGFR were assessed. Only data from outpatient assessments were used in the analysis, but not data from hospitalizations or acute kidney injury events. The analysis included all eGFR measurements recorded between enrollment and the end of the follow-up period. The composite outcomes were primary either a doubling of serum creatinine level or progression to ESKD.

### Statistical analysis

All statistical analyses were performed using GraphPad Software version 9.0, Prism and SPSS (version 22, IBM Inc., Armonk, NY). All *in vitro* experiments were conducted independently and repeated a minimum of three times to ensure consistency and reproducibility of the results. For statistical comparisons between two groups, unpaired two-tailed Student's t-tests were performed. When analyzing more than two groups, one-way Analysis of Variance (ANOVA) was used, followed by Tukey's post hoc test to account for multiple comparisons. For analysis in clinical prospective study, chi-square tests were used for categorical variables and non-normally distributed continuous variables were subjected to log10 transformation to approximate normal distribution. Person-time was calculated from enrollment until the occurrence of kidney outcomes or the end of the study. Kaplan-Meier survival analysis was used to examine association between serum levels of precursor and metabolites in kynurenine pathway (KP) and the cumulative risk of a kidney outcome. Cox proportional hazards analysis was used to examine associations between precursor and metabolites of KP and the primary kidney outcomes. Multivariable models included traditional risk factors of age, sex, comorbidities (hypertension and hyperlipidemia history), medication (use of angiotensin-converting enzyme inhibitors (ACEIs)/angiotensin II receptor blockers (ARBs) or sodium-glucose transport protein 2 inhibitors (SGLT2i)), baseline kidney function (eGFR and log-formed UACR), and glucose control (glycated hemoglobin, HbA1c). A significance level of p-value less than 0.05 was set for all tests.

## Results

### Immune cell infiltration is a hallmark of DN, evident from early to late stages in T2D models

**Figure 1:**
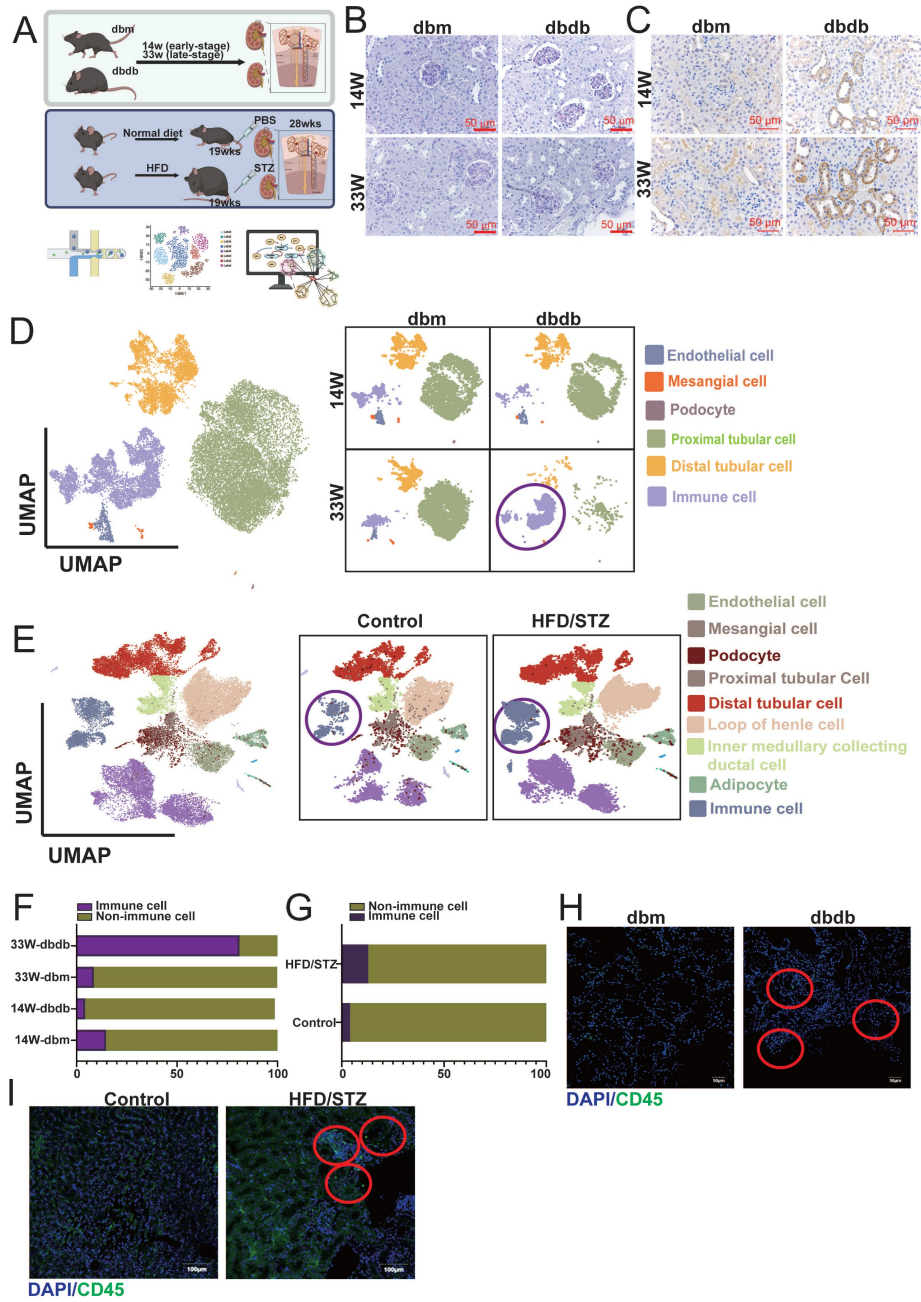
** Characterization of diabetic nephropathy (DN) progression in two mouse models using histology, scRNA-seq, and immunofluorescence. A** Schematic overview of the two mouse models of type 2 diabetes (T2D) used in this study: the transgenic db/db model and the high-fat diet/streptozotocin (HFD/STZ) model (n=3 at each group).** B-C (B)** Periodic acid-Schiff (PAS) and **(C)** KIM-1 staining of kidney sections from db/db mice at early (14 weeks) and late (33 weeks) stages of DN reveals progressive glomerular and tubular injury.** D-E** UMAP plots showing clustering and annotation of major kidney cell types identified by scRNA-seq, including mesangial cells (MCs: ACTA2, PDGFRB), endothelial cells (ECs: PECAM1, CDH5), podocytes (NPHS2, NPHS1), proximal tubule cells (PTCs: SLC34A1), and distal tubule cells (DTCs: SLC12A1, SLC12A3, EPCAM), in both the **(D)** db/db and **(E)** HFD/STZ models. **F-G** Increased immune cell infiltration observed in the diabetic kidneys, as shown by immune cell clusters in the scRNA-seq data from **(F)** db/db and **(G)** HFD/STZ mice.** H-I** Immunofluorescence staining (CD45) confirms enhanced immune cell presence in kidneys from **(H)** db/db and **(I)** HFD/STZ mice.

In our previous studies, we established two mouse models of T2D: the transgenic db/db model 17 and the HFD/STZ model (Figure 1A) 16. Periodic acid-Schiff and KIM-1 staining confirmed that DN begins to develop as early as 14 weeks and becomes more severe by 33 weeks (Figure 1B-C). We collected kidney tissues from the early (14 weeks) and late (33 weeks) stages, as well as from the HFD/STZ model, and performed single-cell RNA sequencing (scRNA-seq) to investigate the mechanisms and progression of DN. UMAP plots revealed that major kidney cell types, including mesangial cells (MCs: ACTA2 (actin alpha 2), PDGFRB (platelet-derived growth factor receptor beta)), endothelial cells (ECs: PECAM1 (platelet and endothelial cell adhesion molecule 1), CDH5 (cadherin 5)), podocytes (NPHS1, NPHS2), proximal tubule cells (PTCs: SLC34A1 (solute carrier family 34 member 1)), distal tubule cells (DTCs: SLC12A1, SLC12A3, EPCAM) and immune cells (PTPRC (protein tyrosine phosphatase receptor type C)), were well preserved and clearly defined in both the transgenic and HFD/STZ models (Figure 1D-E). Interestingly, our results showed a marked increase in immune cell infiltration in the kidneys of both diabetic models (Figure 1F-G). Immunofluorescence staining further confirmed the presence of immune cell infiltration associated with DN (Figure 1H-I) in both diabetic mouse models.

### Chemokine (C-C motif) ligands 4 (CCL4⁺) MDSCs exhibit a pro-inflammatory phenotype and represent the predominant infiltrating immune population in DN

**Figure 2:**
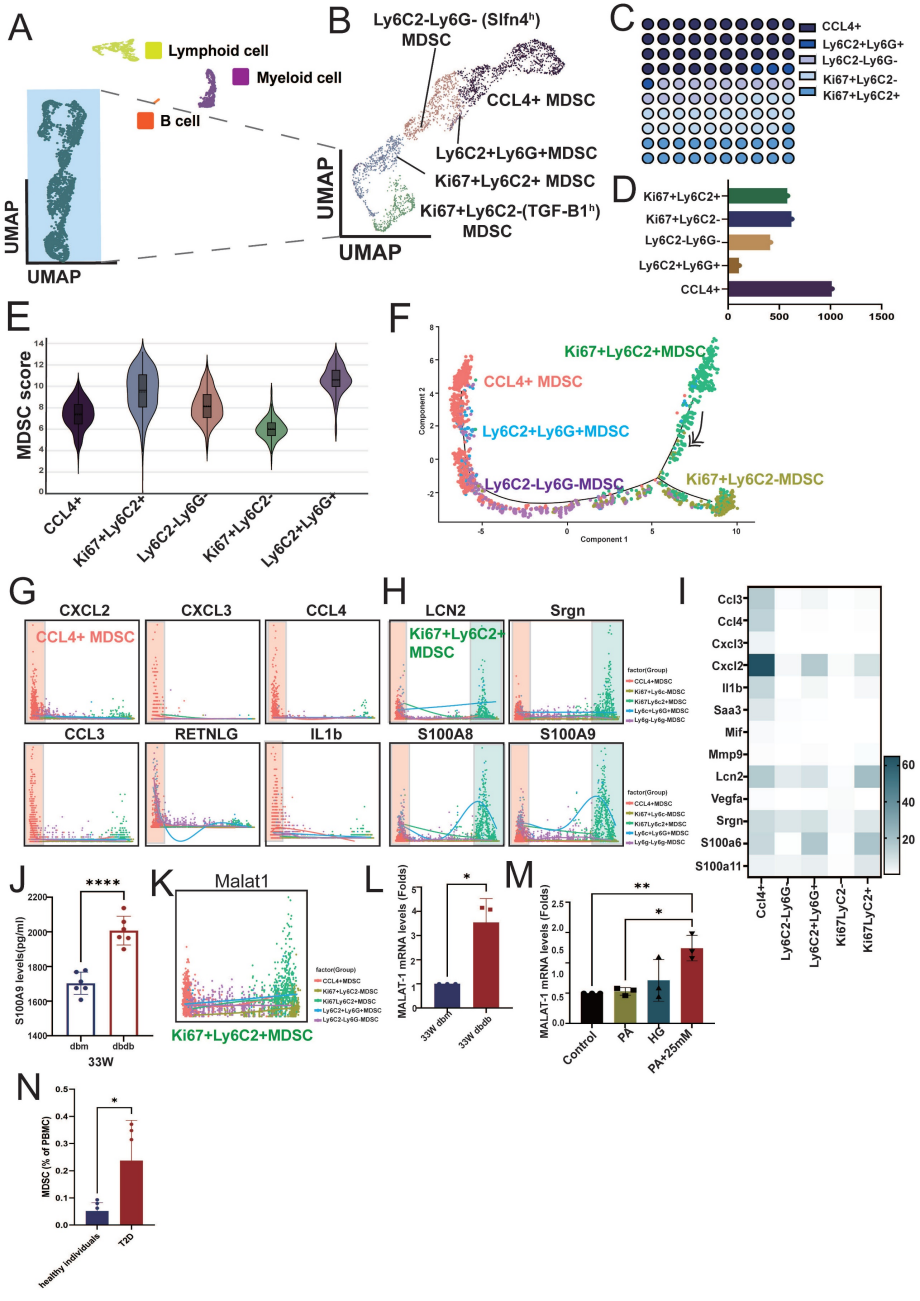
** Characterization and functional profiling of atypical immune cell subsets in diabetic nephropathy. A** Reclustering PTPRC⁺ immune cells identified major immune cell lineages, including lymphoid cells, myeloid cells, B cells, and a distinct population of atypical immune cells from db/m and db/db mice at 14 and 33 weeks. **B** Further subclustering atypical immune cells revealed five myeloid-derived suppressor cell (MDSC) subsets: CC4⁺ cells, LY6C2⁺LY6G⁺ cells, LY6C2⁺LY6G⁻ (SLFN4^h^) cells, MKI67⁺LY6C2⁺ cells, and MKI67⁺LY6C2⁻ (TGFB1^h^) cells. Classification was based on specific markers and proliferation status (MKI67⁺). **C-D** Proportional representation of each atypical subset, with CCL4⁺ cells comprising the most abundant population. **E** MDSC scores across the five subsets, showing highest scores in MKI67⁺LY6C2⁺ and LY6C2⁺LY6G⁺ subsets, and lower scores in CCL4⁺ cells. **F** Pseudotime trajectory analysis revealed that MKI67⁺LY6C2⁺ MDSCs reside at the origin of the differentiation path, while CCL4⁺ MDSCs are positioned at a more differentiated state. **G-H** Expression patterns of representative inflammatory cytokines in MDSCs. CCL4⁺ MDSCs highly expressed **(G)** CXCL2, CXCL3, CCL4, CCL3, RETNLG and IL-1β, while **(H)** LCN2, SRGN, S100A8, and S100A9 were highly expressed in both MKI67⁺LY6C2⁺ and CCL4^+^ MDSCs. **I** The heatmap of inflammatory cytokines across MDSC subsets. **J** S100A9 protein expression was significantly elevated in MDSCs isolated from the kidney of 33-week-old db/db mice (n=6) compared to 33-week-old db/m mice (n=6). **K** The long non-coding RNA MALAT1 was broadly expressed across MDSC subsets. **L** MALAT1 mRNA expression was significantly elevated in MDSCs isolated from 33-week-old db/db mice (n=3) compared to 33-week-old db/m mice (n=3). **M** High glucose (HG) and palmitic acid (PA) stimulation of splenic progenitor cells from normal mice led to increased MALAT1 expression (n=3 at each group). **N** Elevated levels of circulating MDSCs in peripheral blood of T2D patients. Flow cytometry analysis showing a significant increase in the proportion of circulating MDSCs in the peripheral blood of patients with T2D (n=5) compared to healthy controls (n=6). ^*^, p < 0.05, ^**^, p < 0.01, ^***^, p < 0.001, ^****^, p < 0.0001, ns, no significant. Expression values are presented as mean ± SD.

We reclustered the immune cell population (PTPRC⁺ cells) and classified them into lymphoid cells, myeloid cells, B cells, and a group of atypical immune cells based on their cell markers and proliferation status (MKI67⁺, marker of proliferation Ki-67) (Figure 2A). Further analysis of the atypical immune cells revealed five distinct subsets, including CCL4⁺ cells, LY6C2⁺LY6G⁺ cells, LY6C2⁺LY6G⁻ (SLFN4^high(h)^, schlafen 4) cells, MKI67⁺LY6C2⁺ cells, and MKI67⁺LY6C2⁻ (SLFN4^high(h)^) cells (Figure 2B, S1A), with CCL4⁺ immune cells representing the most abundant population (Figure 2C-D). Because this immune population expressed only a subset of canonical immune cell markers, it likely represents incompletely differentiated immune cells, as is well recognized for MDSCs. Therefore, we applied an established MDSC gene signature to calculate an MDSC score for these subsets 19. The result indicated that these subsets exhibited varying levels of myeloid-derived suppressor cell (MDSC) scores (Table S2), with MKI67⁺LY6C2⁺ (proliferative subsets) and LY6G⁺LY6C2⁺ cells showing the highest MDSC scores, whereas CCL4⁺ and MKI67⁺LY6C2^-^ (proliferative subsets) cells had relatively lower scores (Figure 2E). Trajectory analysis supported CCL4⁺ MDSCs represent a more differentiated population, positioning MKI67⁺LY6C2⁺ MDSCs at the origin of the differentiation path and CCL4⁺ MDSCs at a more differentiated state (Figure 2F). Several inflammatory cytokines, including CXCL2, CXCL3, CCL3, CCL4, IL-1β, and RETNLG (resistin like gamma), were highly expressed in CCL4⁺ MDSCs, whereas neutrophil/myeloid inflammatory (alarmin) signature (LCN2, SRGN, S100A8, and S100A9) were highly expressed in both MKI67⁺LY6C2⁺ and CCL4⁺ MDSCs (Figure 2G-I, S1B). These findings indicate that distinct MDSC subsets secrete different cytokines to modulate immune responses. Notably, S100A9 showed the highest expression, which was also confirmed in MDSCs isolated from the kidneys of 33-week-old db/db mice (Figure 2J). Interestingly, the long non-coding RNA MALAT1 was highly expressed across MDSC populations. Given that MALAT1 inhibition has been reported to reduce the MDSC population 20, these findings suggest a potential role for MALAT1 in maintaining MDSC function. (Figure 2K, S1C). Upon isolating MDSCs from 33-week-old db/m and db/db mice, MALAT1 expression was found to be significantly elevated in MDSCs from db/db mice (Figure 2L). Moreover, stimulation of splenic progenitor cells from normal mice under HG and PA conditions led to increased MALAT1 expression (Figure 2M). Most importantly, compared to healthy individuals, T2D patients also exhibited elevated levels of circulating MDSCs in peripheral blood (Figure 2N). These results suggest that MALAT1 may play a role in maintaining MDSCs in the context of DN.

### Lipid-associated macrophages with inflammatory signatures infiltrate diabetic kidneys at late-stage

**Figure 3:**
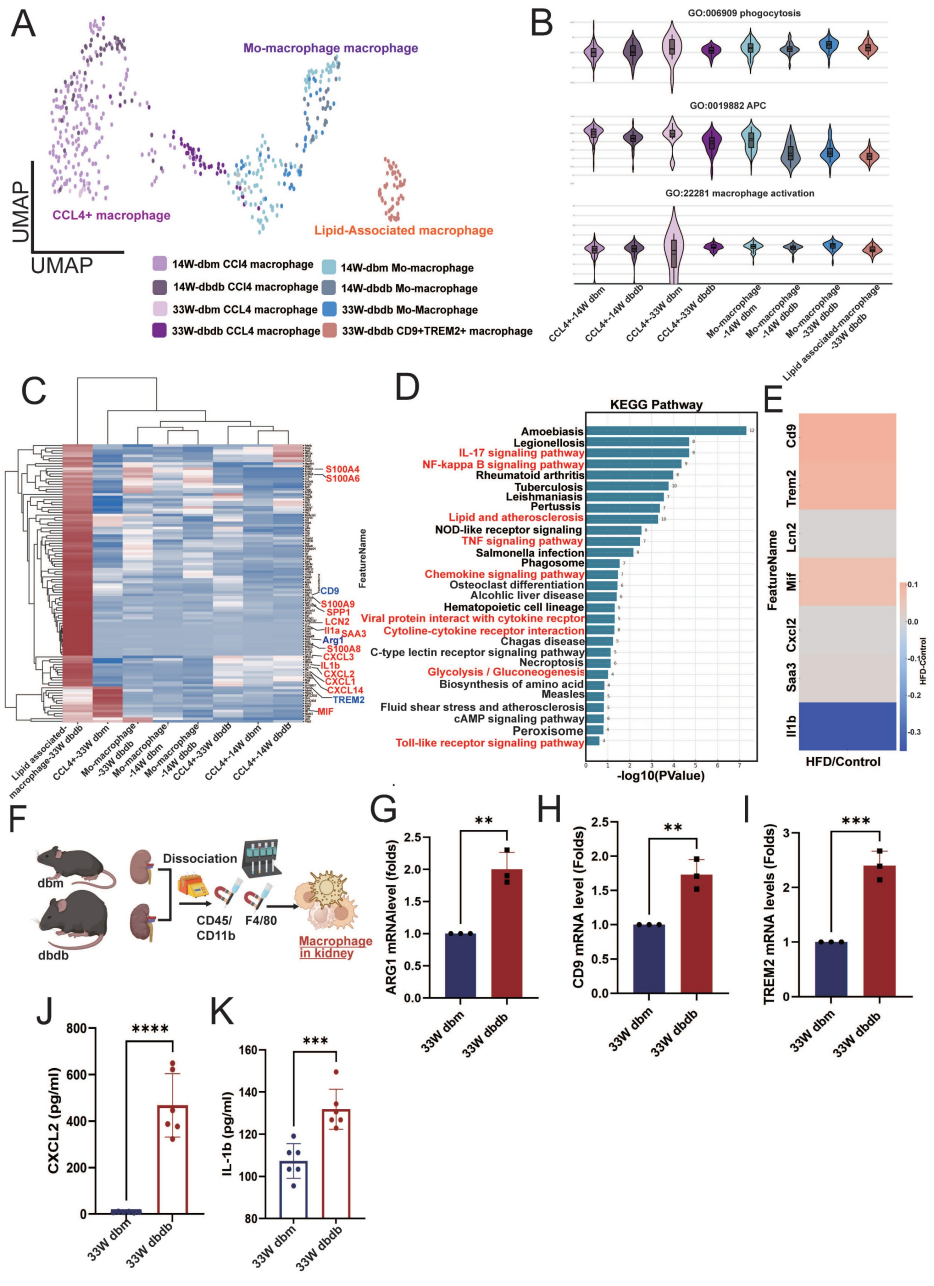
** Lipid-associated macrophages emerge in diabetic kidneys and exhibit enhanced inflammatory and metabolic activation. A** Reclustering CD68⁺ kidney macrophages from db/m and db/db mice at 14 and 33 weeks. **B** Functional scoring of macrophages revealed reduced phagocytic activity, antigen-presenting cell (APC) capacity, and activation status in 14-week db/db kidneys, with a marked decrease in these functions by 33 weeks. **C** Differential gene expression (DEG) analysis of lipid associated macrophages from 33-week-old db/db kidneys showed upregulation of lipid-associated markers (CD9, TREM2) and pro-inflammatory genes (SAA3, S100A4, S100A8, S100A9, SPP1, CXCL2, MIF, and IL-1β). **D** KEGG pathway enrichment analysis of lipid-associated macrophages from 33-week-old db/db kidneys indicated enhanced activity in glucose and lipid metabolism-related pathways. **E** Expression of CD9, TREM2, CXCL2, MIF, and SAA3 was similarly elevated in kidney-infiltrating macrophages from HFD/STZ-induced diabetic mice. **F** Schematic overview of kidney macrophage isolation. **G-I** qPCR confirmed increased expression of the M2 macrophage marker **(G)** ARG1, **(H)** CD9, and **(I)** TREM2 in kidney macrophages from 33-week db/db mice (n=3 at each group). **J-K** Elevated secretion of inflammatory cytokines **(J)** CXCL2 and **(K)** IL-1β was detected in macrophages isolated from 33-week db/db kidneys (n=6 at each group); quantification confirms significantly higher levels compared to controls. ^*^, p < 0.05, ^**^, p < 0.01, ^***^, p < 0.001, ^****^, p < 0.0001, ns, no significant. Expression values are presented as mean ± SD.

Macrophage infiltration has been implicated in the pathogenesis of DN 21, so we further analyzed CD68⁺ macrophages (Figure 3A). We identified three subsets: CCL4⁺ macrophages (CCL4⁺), monocyte-derived macrophages (PLAC8⁺, Placenta Associated 8), and lipid-associated macrophages (CD9⁺/TREM2⁺, Triggering receptor expressed on myeloid cells 2) 22,23. Interestingly, lipid-associated macrophages were observed only in kidneys from 33-week-old db/db mice (Figure 3A). Functionally, monocyte-derived macrophages in early-stage diabetic kidneys (14 weeks) showed reduced antigen-presenting cell (APC) capacity relative to macrophages in db/db kidneys. By 33 weeks, however, both monocyte-derived and lipid-associated macrophages infiltrating db/db kidneys exhibited a significant decrease in APC capacity, while phagocytosis and activation showed no obvious differences (Figure 3B). Differential gene-expression analysis revealed upregulation of lipid-associated markers (CD9 and TREM2), M2 type-macrophage marker (ARG1) and multiple inflammatory mediators including SAA3 (serum amyloid A3), S100A4, S100A6, S100A8, S100A9, SPP1, CXCL1/2/3, CXCL14, LCN2, and IL-1α/β in kidney lipid-associated macrophages from 33-week-old db/db mice (Figure 3C). KEGG pathway enrichment further highlighted the involvement of glucose and lipid metabolic pathways in lipid associated macrophages from db/db kidneys at 33 weeks (Figure 3D). HFD/STZ model similarly demonstrated elevated expression of CD9, TREM2, CXCL2, MIF (macrophage migration inhibitory factor), and SAA3 in kidney-infiltrating macrophages (Figure 3E). In situ analysis confirmed increased expression of the M2 marker ARG1, and lipid associated macrophage markers CD9, and TREM2 in kidney macrophages isolated from 33-week-old db/db mice (Figure 3F-I). The secreted elevated levels of CXCL2 and IL-1β were also found in macrophages from kidney of 33-week-old db/db mice (Figure 3J-K). These findings suggest that lipid-associated macrophages are a key infiltrating population in the diabetic kidney and may contribute to the progression of DN.

### Temporal dynamics of serum inflammatory factors during DN progression

Since both macrophages and MDSCs infiltrating diabetic kidneys exhibited high expression of various secreted inflammatory factors, we further investigated the temporal dynamics of these molecules. In the serum of diabetic mice, levels of CXCL2 and S100A9 began to increase during the early stage of DN (Figure S2A-B), while SRGN expression rose during the mid-stage, and LCN2 levels were predominantly elevated in the late stage of DN (Figure S2C-D). In contrast, serum IL-1β levels did not show statistically significant changes at any stage (Figure S2E). These findings suggest that CXCL2 and S100A9 may serve as potential early biomarkers for the prediction and monitoring of DN progression.

### GPX3 and SPP1-associated endothelial reprogramming in diabetic kidneys

**Figure 4:**
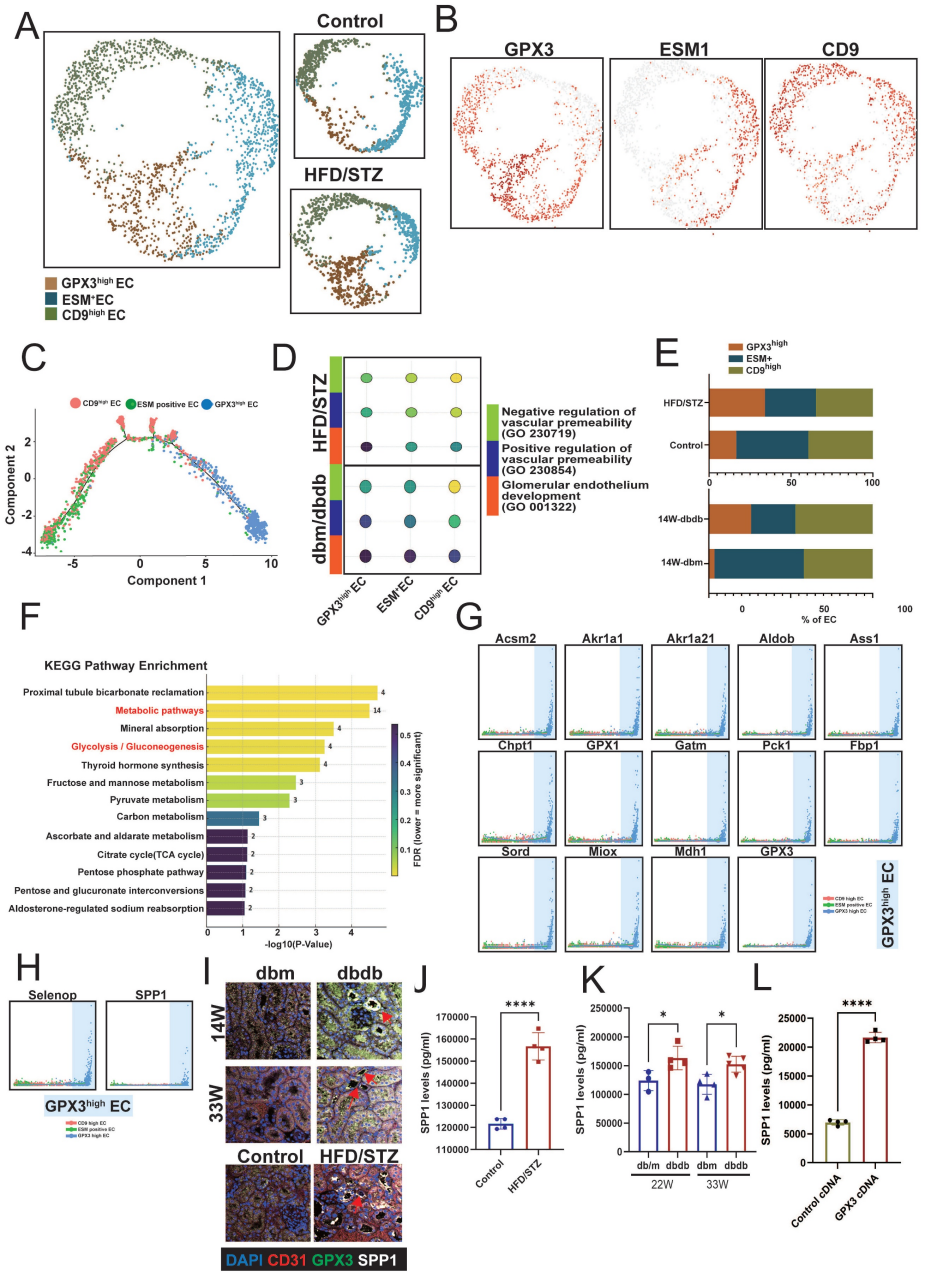
** Characterization of renal endothelial cell (EC) subsets and their association with diabetic nephropathy (DN) progression. A** UMAP plot showing clustering renal ECs from the HFD/STZ mouse model into three subsets based on marker gene expression: GPX3^high^, ESM1⁺, and CD9^high^ ECs. **B** Feature plots showing the expression levels of representative marker genes for each EC subset. **C** Trajectory analysis indicating a differentiation path from GPX3^high^ (less differentiated) to CD9^high^ (terminally differentiated) ECs. **D** Functional scoring of EC subsets showing CD9^high^ ECs possess the highest glomerular endothelial identity and vascular permeability, while GPX3^high^ ECs exhibit the lowest scores. **E** The proportions of EC subsets in diabetic versus control mice. **F** KEGG pathway enrichment analysis of differentially expressed genes in GPX3^high^ ECs, highlighting significant enrichment in metabolic pathways. **G** The involvement of metabolic genes in transitions between EC subsets. **H** Identification of SELENOP and SPP1 as soluble factors associated with GPX3^high^ EC formation. **I**. Immunofluorescence staining revealed the GPX3^+^SPP1^+^ ECs in peritubular capillaries (arrow) of the kidney of two diabetic mouse models. **J-K** ELISA quantification of SPP1 levels in serum from **(J)** HFD/STZ and **(K)** db/db mice at mid- and late-stage DN (n=4 and 5 respectively), confirming elevated SPP1 in disease states. **L** Overexpression of GPX3 increased SPP-1 expression in GECs (n=4). ^*^, p < 0.05, ^**^, p < 0.01, ^***^, p < 0.001, ^****^, p < 0.0001, ns, no significant. Expression values are presented as mean ± SD.

Changes in renal vasculature, particularly glomerular endothelial alterations, are key indicators of kidney disease progression 24. Therefore, we next focused on analyzing changes in ECs. Given the higher number of ECs captured in the HFD/STZ model, we performed EC clustering using this T2D model. Based on the expression of EC marker genes, renal ECs were classified into three subsets: GPX3^high^, ESM1^+^ (endothelial cell-specific molecule 1), and CD9^high^ ECs (Figure 4A-B). Trajectory analysis revealed that GPX3^high^ ECs represent a less differentiated state, whereas CD9^high^ ECs are positioned at the terminal end of the differentiation trajectory (Figure 4C). Functional analysis supported this observation, showing that CD9^high^ ECs had the highest scores for glomerular endothelial identity and vascular permeability, while GPX3^high^ ECs exhibited lower scores (Figure 4D). When comparing the distribution of EC subsets in diabetic mice, we observed an increased proportion of GPX3^high^ ECs and a marked decrease in CD9^high^ ECs of HFD/STZ mice (Figure 4E). KEGG pathway analysis of the DEGs in GPX3^high^ ECs revealed enrichment in metabolic pathways, including mineral absorption, glycolysis, carbon metabolism, and pyruvate and fructose metabolism (Figure 4F). These metabolic genes were also associated with transitions among the three EC subsets, skewing toward the GPX3^high^ EC state (Figure 4G). Two soluble factors—SELENOP (selenoprotein P) and SPP1—were identified as contributors to the formation of GPX3^high^ ECs (Figure 4H). Furthermore, IF showed that ECs of peritubular capillaries in the kidney co-expressed GPX3 and SPP1 (Figure 4I). ELISA confirmed elevated SPP1 levels in both the HFD/STZ model and db/db mice at the mid and late stages of DN (Figure 4J-K). Overexpression of GPX3 increased the expression of SPP1 in GECs (Figure 4L). Our findings suggest that ECs with high GPX3 and SPP1 expression are associated with the progression of DN.

### Identification of upregulated kynureninase (KYNU) in early PT injury

**Figure 5:**
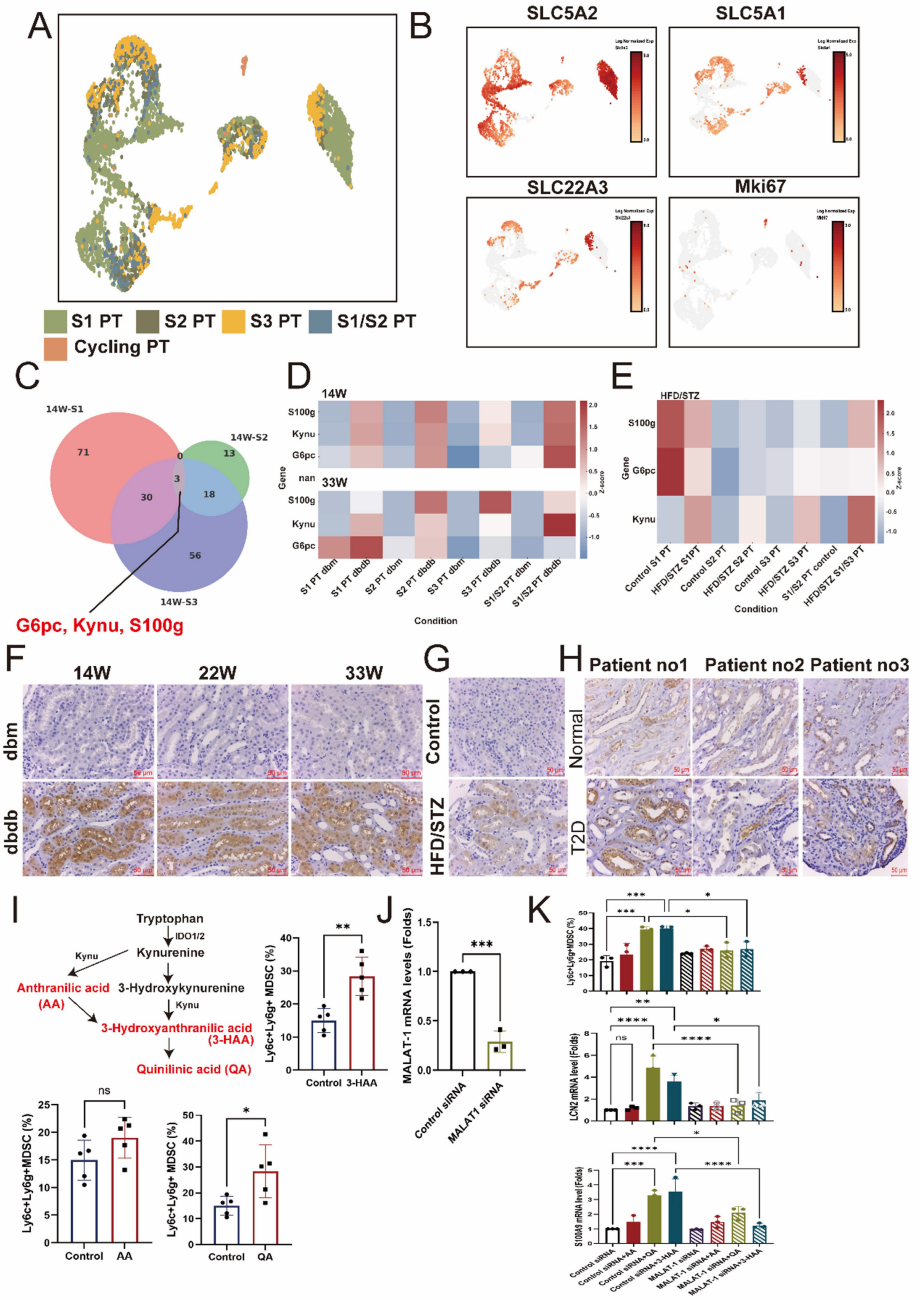
** Identification of early injury markers in proximal tubule (PT) cells during diabetic nephropathy (DN). A** UMAP plot showing the classification of PT cells into S1, S2, S3, S1/2 (SLC5A1/2 double-positive), and cycling PT subsets based on marker gene expression. **B** Feature plots depicting expression levels of SLC5A2 (S1 PT), SLC5A1 (S2 PT), SLC22A3 (S3 PT), and MKi67 (cycling cells). **C** Venn diagram showing the intersection of upregulated genes in S1, S2, and S3 PTs, identifying KYNU, S100G, and G6PC as common markers of early PT injury. **D-E** Heatmap illustrating increased expression of Kynu, S100g, and G6PC in PT subtypes of diabetic mice (db/db model and HFD/STZ model) compared to db/m and wild-type controls. **F** Representative immunohistochemistry (IHC) images showing elevated kynureninase expression in PT cells of db/db mice relative to db/m mice at 14, 22 and 33 weeks. **G** IHC staining in the HFD/STZ model confirming increased kynureninase expression in PT cells of diabetic mice. **H** Elevated kynureninase (KYNU) expression in the PT cells of DN compared to those of normal part of upper tract urothelial carcinoma (n=3 at each group). **I** Kynureninase-derived metabolites promote MDSC generation from mouse bone marrow cells. Mouse bone marrow cells were cultured in the presence of 3-hydroxyanthranilic acid (3-HAA, 50 μM) and quinolinic acid (QA, 50 μM). Flow cytometry analysis showed an increased proportion of CD11b⁺Ly6C⁺Ly6G⁺ myeloid-derived suppressor cells (MDSCs) compared to control conditions (n=5 at each group). **J** Knockdown efficiency of MALAT11 siRNA in mice bone marrow cells (n=3). **K** Silencing MALAT1 abrogated QA- and 3-HAA-induced increases in increase of CD11b⁺Ly6C⁺Ly6G⁺ MDSC, and LCN2 and S100A9 expression (n=3 at each group). ^*^, p < 0.05, ^**^, p < 0.01, ^***^, p < 0.001, ^****^, p < 0.0001, ns, no significant. Expression values are presented as mean ± SD.

Next, we analyzed changes in the proximal tubule (PT), classifying the S1, S2, and S3 segments based on the expression of SLC5A2 (S1 PT), SLC5A1 (S2 PT), SLC22A3 (S3 PT), and MKI67 to identify S1, S2, S3, S1/2 (SLC5A1/2 double-positive), and cycling PT cell populations in the transgenic db/db model (Figure 5A-B). To identify genes commonly involved in early PT injury, we intersected the upregulated genes in S1, S2, and S3 PT cells and found that KYNU, S100g, and G6PC (glucose-6-phosphatase catalytic subunit 1) were consistently overexpressed (Figure 5C). Furthermore, these three genes were also upregulated in the late stage of DN and across various PT subtypes in kidneys from the HFD/STZ model, compared to db/m and wild-type control mice, respectively (Figure 5D-E). IHC stain confirmed that the expression of KYNU was significantly elevated in PT cells of db/db mice compared to db/m mice, with consistent findings observed at 14, 22 and 33 weeks (Figure 5F). A similar upregulation of KYNU was also observed in the PT cells in the HFD/STZ model (Figure 5G). Importantly, in human kidney biopsy samples, KYNU expression was markedly increased in PT cells of patients with DN compared to individuals with normal renal function (Figure 5H). Furthermore, downstream metabolites of KYNU, including 3-HAA and QA, promoted the differentiation of mouse bone marrow cells into CD11b⁺LY6C⁺LY6G⁺ MDSCs (Figure 5I). Notably, MALAT1 knockdown markedly attenuated the ability of 3-HAA and QA to drive this differentiation, and LCN2 and S100A9 upregulation (Figure 5J-K), indicating their role in promoting MDSC transition and inflammation.

### Metabolites of kynurenine pathway (KP) and adverse kidney outcomes in T2D patients

As KYNU involved in the metabolism of KP that has been associated with immune modulation 25, we further examined circulating levels of precursor and metabolites of KP, including tryptophan, kynurenine, KA, 3-HK, QA, and XA in 497 T2D patients. The mean age and T2D duration were 60.7 years-old and 9.3 years respectively, and 53.5 % were male. The prevalence rates of hypertension and hyperlipidemia were 57.1 % and 69.3 % respectively. The mean eGFR was 81.2 ml/min/1.73 m^2^, and the median HbA1C and UACR were 7.0 % and 16.6 mg/g respectively. The median serum concentration of tryptophan, the precursor of KP, was 55.40 μM, while the median levels of KP metabolites were 1.32 μM for kynurenine, 0.06 μM for KA, 0.04 μM for 3-HK, 0.43 μM for QA, and 0.30 μM for XA (Table S3).

**Figure 6:**
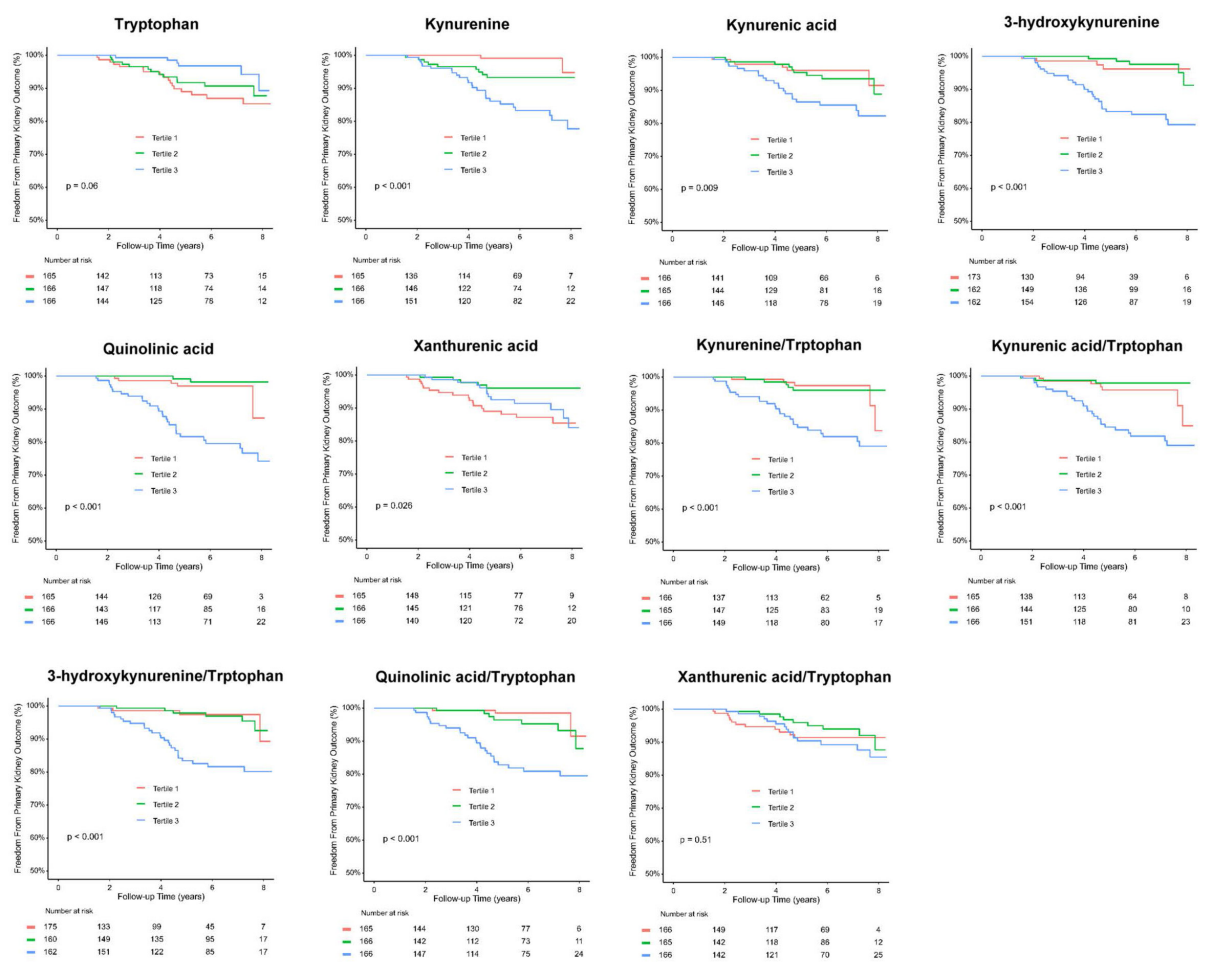
Kaplan-Meier survival analysis for evaluation of the relationship between primary kidney outcomes of either doubling serum creatinine levels or progression to end stage kidney disease and tertiles of serum kynurenine, kynurenic acid, 3-hydroxykynurenine, quinolinic acid, xanthurenic acid, kynurenine/tryptophan, kynurenic acid/tryptophan, 3-hydroxykynurenine/tryptophan, quinolinic acid/tryptophan, and xanthurenic acid/tryptophan in type 2 diabetic patients.

Over a mean follow-up period of 5.3 years, 36 patients (7.2 %) reached doubling of serum creatinine levels or progression to ESKD, 35 patients (7.0 %) reached doubling of serum creatinine levels, 13 patients (2.6%) developed ESKD, and 31 patients (6.2 %) died (Table 1). Kaplan-Meier curves showed a significantly higher cumulative incidence of composite kidney outcomes (ESKD or doubling of serum creatinine levels) among T2D patients across tertile 1 to 3 of serum kynurenine, KA, 3-HK, QA, kynurenine/tryptophan, KA/tryptophan, 3-HK/tryptophan, and QA/tryptophan levels (Figure 6). Univariate Cox analysis was performed to ascertain associations between the tertiles of serum precursor and metabolites levels of KP and the composite kidney outcomes. Compared to tertile 1, tertile 3 of serum kynurenine, KA 3-HK, QA, kynurenine/tryptophan, KA/tryptophan, 3-HK/tryptophan, and QA/tryptophan was significantly associated with increased risk for kidney outcomes. Conversely, tryptophan was negatively correlated with kidney outcomes (Table 1). After adjusting for traditional risk factors for adverse kidney outcomes including age, sex, hypertension, hyperlipidemia, ACEIs/ ARBs or SGLT2 inhibitor usage, HbA1C, baseline eGFR at 60 ml/min/1.73m^2^, log form urinary ACR, the patients in highest tertile of serum kynurenine, 3-HK, and QA had increased risk for kidney outcomes compared to those in lowest tertile of serum kynurenine (hazard ratio (HR): 5.46, 95% confidence index (CI): 1.18-25.30, p=0.03), 3-HK (HR: 4.91, 95% CI: 1.40-17.20, p=0.01), QA (HR: 3.42, 95% CI: 1.26-9.31, p=0.02), kynurenine/tryptophan (HR: 2.88, 95% CI: 1.01-8.19, p=0.04), 3-HK/tryptophan (HR: 4.80, 95% CI: 1.37-16.75, p=0.01), and QA/tryptophan (HR: 5.63, 95% CI: 1.62-19.55, p=0.006) (Table 1). The reverse relationship between serum tryptophan and adverse kidney outcomes was consistent in adjusted model (HR: 0.30, 95% CI: 0.11-0.82, p=0.02). However, there was no significant relationship between kidney outcomes and KA, XA, KA/tryptophan and XA/tryptophan. These findings suggest KP precursor tryptophan and KP metabolites, especially kynurenine, 3-HK, and QA have the potential to predict adverse kidney outcomes in T2D patients.

## Discussion

**Figure 7:**
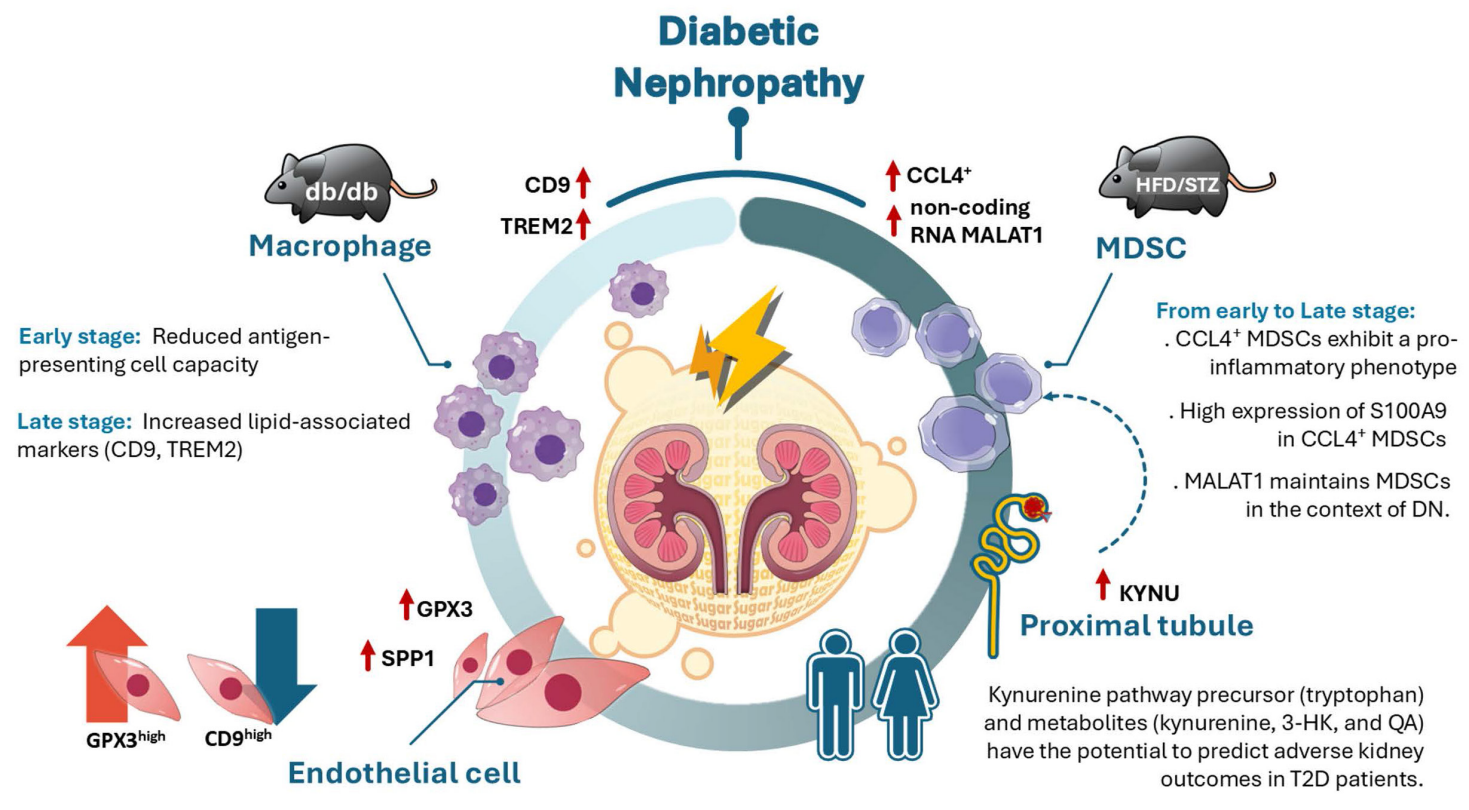
Illustration of the mechanism of dynamic immune-inflammatory landscape of DN from early to late stage. CCL4⁺ myeloid-derived suppressor cells (MDSCs) with a pro-inflammatory phenotype emerged as the predominant infiltrating immune population in DN progression, with S100A9 highly expressed in MDSCs. The long non-coding RNA MALAT1 was identified as a key factor in maintaining MDSC function. In the early stage of DN, antigen-presenting cell activity was decreased, while in the late stage of DN, elevated expression of CD9 and TREM2 in kidney macrophages suggested a role for lipid-associated macrophages in DN progression. Endothelial cell reprogramming characterized by GPX3 and SPP1 expression was observed during DN advancement. Furthermore, upregulation of KYNU, a key enzyme in the kynurenine pathway (KP), was detected in proximal tubule injury during early stage of DN, while KP metabolites including tryptophan, kynurenine, 3-hydroxykynurenine, and quinolinic acid have the potential to predict adverse renal outcomes in T2D patients.

The study integrated scRNA-seq, in vivo model, and clinical T2D patients to explore the dynamic cellular changes in different stages of DN, and showed a marked increase in immune cell infiltration in the early stage of DN of two models. CCL4⁺ MDSCs exhibit a pro-inflammatory phenotype and represent the predominant infiltrating immune population in DN from early to late stage. S100A9 was highly expressed in CCL4⁺ MDSCs and also as early indicators of DN in mouse models. Non-coding RNA MALAT1 participated in maintaining MDSCs function in DN. Expression of CD9 and TREM2 were increasing in kidney macrophages obtained from late stage of DN, meaning that lipid-associated macrophages may contribute to DN progression. GPX3 and SPP1-associated endothelial reprogrammed in DN progression. In addition, KYNU as involved in the metabolism of KP, was upregulated in PT Injury from early to late stage of DN. Furthermore, KP precursor tryptophan and KP metabolites, especially, kynurenine, 3-HK, and QA, have the potential to predict adverse kidney outcomes in clinical T2D patients (Figure 7).

Our study reveals that CCL4⁺ MDSCs are the predominant immune cell subset infiltrating the diabetic kidney, exhibiting an inflammatory phenotype and expressing high levels of cytokines such as CXCL2, CXCL3, CCL3, CCL4, and S100A family proteins. While these cells display relatively lower MDSC scores compared to proliferative KI67⁺LY6C2^⁺/⁻^ subsets, their abundance and inflammatory signature suggest a distinct functional state within the microenvironment of the diabetic kidney. Importantly, inflammatory mediators including LCN2, S100A9, and CXCL2 were elevated in the serum of diabetic mice, with CXCL2 and S100A9 further upregulated during the early stage of DN, reinforcing their potential involvement in DN pathogenesis. Notably, the long non-coding RNA MALAT1 is highly expressed across MDSC populations and is further upregulated in diabetic mice and under metabolic stress conditions, suggesting a role in maintaining MDSC function in this context. Previous studies have confirmed that MALAT1 expression is elevated in peripheral blood mononuclear cells from diabetic patients 26, 27, and in mouse models 28. MALAT1 has been associated with the progression of DN. Additionally, MALAT1 has been shown to regulate MDSC expansion via the STAT3 signaling pathway, and inhibition of MALAT1 can reduce MDSC population 29, 30. Although our findings suggest that MALAT1 may contribute to sustaining or shaping the inflammatory profile of MDSCs in the diabetic kidney, its precise regulatory mechanisms remain unclear. Whether MALAT1 directly affects MDSC lineage commitment, phenotypic stability, or cytokine expression patterns under hyperglycemic and lipotoxic stress requires further mechanistic investigation to elucidate its role in chronic kidney inflammation during T2D.

With the advancement of scRNA-seq technology, a wide variety of immune cell subsets have been identified across different diseases, including distinct macrophage populations 31. Several studies have consistently reported the presence of macrophage subsets characterized by high expression of lipid-associated genes such as CD9, TREM2, CD36, and APOE 32, 33. These lipid-associated macrophages have been implicated in the pathogenesis of various diseases, including cancer, endometriosis and fibrosis 34. TREM2^high^ macrophages reside in the human adult kidney and TREM2^high^ macrophages increase in frequency in a HFD mouse model and human with DN 35, 36. Subramanian et al. reported that knockout of TREM2 in mice with a high-fat diet exhibit exacerbated glomerular damage and increased tubular epithelial cell injury, suggesting that TREM2^high^ macrophages may play a critical protective role in maintaining kidney injury under metabolic stress conditions 36. In our findings, we observed that the infiltration of CD9^high^TREM2^high^ macrophages primarily appear in the late stage (33W) of DN in mice, but not in the early stage (14W) of DN. However, when comparing TREM2^+^ and TREM2^-^ macrophages, we found that the latter exhibited higher expression of CCR2 (data not shown), which has been implicated in monocyte/macrophage-induced kidney injury in diabetes 37. These results suggest that TREM2^-/low^ macrophages may play a detrimental role in DN progression, whereas the accumulation of TREM2^+/high^ macrophages in the kidney during late stage raises the possibility that they may serve a protective or compensatory role. Whether they can prevent or mitigate DN development requires further investigation with more detailed time-course studies.

Recent studies have identified PTECs as important regulators of the renal immune microenvironment 38. PTECs exhibit both innate and adaptive immune characteristics, producing proinflammatory cytokines, chemokines, complement components, and even immunoglobulins. Moreover, PTECs have been characterized as renal non-professional APCs capable of inducing antigen-specific CD4⁺ T cell activation. Our findings found that KYNU was dominantly expressed in PT area of DN through early to late stage. KYNU is one of principle enzymes involved in KP as a critical metabolic route for the degradation of 95% ingested tryptophan in mammals. KP has been reported in pathogenesis of several diseases including neurological disorders, mental illnesses, cardiovascular disease, or autoimmune diseases 39, 40. KP activation generates several inflammation-related metabolites that might damage pancreatic β-cells, affecting blood glucose control. In turn, prolonged hyperglycemia results in the aberrant KP activation in pancreatic β-cells 41. Only few studies explored the pathophysiologic role of KP metabolites in kidney diseases 25, 42, 43. The kidney is a primary organ for tryptophan metabolism, and KP was associated with immune and inflammation regulation, oxidative stress, uremic toxin production, and atherosclerosis that are important pathophysiologic mechanisms of kidney diseases. Elevated activities of KP enzymes including kynurenine 3-monooxygenase (KMO), KYNU and indoleamine 2,3-dioxygenase (IDO) were found in various kidney diseases 44. Saliba et al. reported elevated plasma kynurenine metabolites in patients with advanced CKD (stages 3b-5), and QA had potentially linked kidney injury to neurological complications such as uremic encephalopathy 43. The proinflammatory milieu may activate immune cells that subsequently enhancing tryptophan-KP metabolism, such as tryptophan converting into kynurenine and synthesizing QA, further leading to vicious cycle 40, 45. QA accumulation upregulates the expression of chemokines, including stromal cell-derived factor 1α, CXCL9, and CCR5 40. Kynurenine induces the release of cytochrome C and the activation of caspase-3, and suppresses NK cell growth and impairing their function 46. In addition, imbalance of tryptophan-KP metabolism could result in oxidative stress and lipid peroxides 40. 3-HK could generate auto-oxidation products and result ROS formation through interacting with cellular xanthine oxidase, further inducing internucleosomal DNA damage 47. Thus, alteration the balance within KP metabolites possibly affects the onset and progression of DN. We firstly demonstrated that KP metabolites, especially kynurenine, 3-HK, and QA, and imbalance of tryptophan-KP metabolism such as kynurenine/tryptophan, 3-HK/tryptophan, and QA/tryptophan were significantly associated with adverse kidney outcomes in clinical T2D patients during follow-up within five years. Our findings provide novel and translational insights of KP metabolites as potential biomarkers and in pathogenesis of DN progression.

## Conclusions

Collectively, our findings present a high-resolution single-cell atlas of immune-related inflammation across early to late stages of DN. This atlas reveals previously unrecognized alterations in immune cell populations and associated KP metabolites during DN progression, underscoring the complexity and heterogeneity of immune responses and cellular transitions in the disease. Future research to validate the functional implications of the specific transcriptional dysregulations identified in this study may offer deeper insights into the underlying molecular mechanisms and pave the way for novel diagnostic approaches and personalized therapeutic strategies for DN patients.

## Supplementary Material

Supplementary figures and tables.

## Figures and Tables

**Table 1:** The association between serum kynurenine pathway precursor and metabolites and kidney composite outcomes either doubling serum creatinine or end stage kidney disease in patients with type 2 diabetic patients

	Event N (%)	Follow-up period (years)	Event per 1000 patient-year	Crude HR (95% CI)	P-value	Adjusted HR^1^ (95%CI)	P-value
**ESKD or doubling of serum creatinine**	36/497 (7.2)	5.3±2.2					
**Serum tryptophan**							
Tertile 1	17/165 (10.3)	5.2±2.3	19.6	1.00 (Reference)		1.00 (Reference)	
Tertile 2	13/166 (7.8)	5.3±2.3	14.6	0.74 (0.36-1.52)	0.41	0.75 (0.35-1.61)	0.46
Tertile 3	6/166 (3.6)	5.3±2.1	6.77	**0.34 (0.13-0.86)**	**0.02**	**0.30 (0.11-0.82)**	**0.02**
**Serum kynurenine**							
Tertile 1	2/165 (1.2)	5.1±2.3	2.3	1.00 (Reference)		1.00 (Reference)	
Tertile 2	9/166 (5.4)	5.2±2.2	10.2	4.25 (0.92-19.67)	0.06	1.98 (0.40-9.91)	0.40
Tertile 3	25/166 (15.1)	5.5±2.2	27.2	**11.26 (2.66-47.57)**	**0.001**	**5.46 (1.18-25.30)**	**0.03**
**Serum kynurenic acid**							
Tertile 1	6/166 (3.6)	5.0±2.3	7.2	1.00 (Reference)		1.00 (Reference)	
Tertile 2	9/165 (5.5)	5.5±2.1	9.9	1.31 (0.47-3.67)	0.61	0.59 (0.19-1.84)	0.36
Tertile 3	21/166 (12.7)	5.4±2.2	27.2	**3.14 (1.27-7.78)**	**0.01**	0.89 (0.31-2.57)	0.82
**Serum 3-hydroxykynurenine**							
Tertile 1	3/162 (1.9)	4.3±2.3	4.2	1.00 (Reference)		1.00 (Reference)	
Tertile 2	5/162 (3.1)	6.0±2.0	5.1	1.05 (0.25-4.40)	0.94	2.04 (0.45-9.21)	0.35
Tertile 3	27/162 (16.7)	5.7±2.0	29.2	**6.17 (1.87-20.36)**	**0.003**	**4.91 (1.40-17.20)**	**0.01**
**Serum quinolinic acid**							
Tertile 1	5/165 (3.0)	5.3±2.0	5.6	1.00 (Reference)		1.00 (Reference)	
Tertile 2	2/166 (1.2)	5.4±2.3	2.2	0.40 (0.08-2.05)	0.39	0.31 (0.06-1.77)	0.18
Tertile 3	29/166 (17.5)	5.0±2.3	33.4	**5.97 (2.30-15.50)**	**< 0.001**	**3.42 (1.26-9.31)**	**0.02**
**Serum xanthurenic acid**							
Tertile 1	18/165 (10.9)	5.3±2.2	20.3	1.00 (Reference)		1.00 (Reference)	
Tertile 2	5/166 (3.0)	5.3±2.2	5.7	0.27 (0.10-0.74)	0.01	0.25 (0.09-0.72)	0.01
Tertile 3	13/166 (7.8)	5.3±2.3	14.9	0.71 (0.35-1.45)	0.34	0.58 (0.26-1.30)	0.18
**Serum kynurenine/tryptophan**							
Tertile 1	5/166 (3.0)	5.0±2.2	6.0	1.00 (Reference)		1.00 (Reference)	
Tertile 2	5/165 (3.0)	5.5±2.2	5.5	0.88 (0.25-3.03)	0.83	0.81 (0.22-2.93)	0.74
Tertile 3	26/166 (15.7)	5.4±2.2	28.7	**4.66 (1.78-12.15)**	**0.002**	**2.88 (1.01-8.19)**	**0.04**
**Serum kynurenic acid/tryptophan**							
Tertile 1	7/165 (4.2)	5.0±2.3	8.4	1.00 (Reference)		1.00 (Reference)	
Tertile 2	3/166 (1.8)	5.4±2.2	3.3	0.40 (0.10-1.53)	0.18	0.41 (0.10-1.74)	0.22
Tertile 3	26/166 (15.7)	5.5±2.2	28.4	**3.33 (1.44-7.68)**	**0.005**	1.73 (0.69-4.32)	0.24
**Serum 3-hydroxykynurenine /tryptophan**							
Tertile 1	3/163 (1.8)	4.4±2.3	4.1	1.00 (Reference)		1.00 (Reference)	
Tertile 2	6/161 (3.8)	6.0±1.9	6.2	1.35 (0.34-5.41)	0.67	2.32 (0.54-10.10)	0.26
Tertile 3	26/162 (16.0)	5.6±2.1	28.5	**6.36 (1.92-21.05)**	**0.002**	**4.80 (1.37-16.75)**	**0.01**
**Serum quinolinic acid/tryptophan**							
Tertile 1	3/166 (1.8)	5.4±2.0	3.3	1.00 (Reference)		1.00 (Reference)	
Tertile 2	7/166 (4.2)	5.1±2.3	8.2	2.59 (0.67-10.01)	0.16	2.57 (0.64-10.27)	0.18
Tertile 3	26/166 (15.7)	5.3±2.3	29.1	**8.97 (2.71-29.72)**	**< 0.001**	**5.63 (1.62-19.55)**	**0.006**
**Serum xanthurenic acid/tryptophan**							
Tertile 1	12/166 (7.2)	5.2±2.0	13.8	1.00 (Reference)		1.00 (Reference)	
Tertile 2	9/165 (5.5)	5.3±2.3	10.1	0.72 (0.30-1.71)	0.45	1.14 (0.43-3.05)	0.78
Tertile 3	15/166 (9.0)	5.3±2.3	16.9	1.17 (0.54-2.51)	0.68	1.43 (0.61-3.35)	0.40

Abbreviations: ESKD, end stage kidney disease; eGFR, estimated glomerular filtration rate; HR, hazard ratio; CI, Confidence interval. ^1^The multivariable model was adjusted for age, sex, hypertension, hyperlipidemia, ACEI/ARB usage, SGLT2 inhibitor usage, HbA1C, baseline eGFR at 60 ml/min/1.73m^2^, log form urinary albumin/creatinine ratio

## References

[1] Ma X, Liu R, Xi X, Zhuo H, Gu Y (2025). Global burden of chronic kidney disease due to diabetes mellitus, 1990-2021, and projections to 2050. Front Endocrinol (Lausanne).

[2] Farah RI, Al-Sabbagh MQ, Momani MS, Albtoosh A, Arabiat M, Abdulraheem AM (2021). Diabetic kidney disease in patients with type 2 diabetes mellitus: a cross-sectional study. BMC Nephrology.

[3] Hall ME, do Carmo JM, da Silva AA, Juncos LA, Wang Z, Hall JE (2014). Obesity, hypertension, and chronic kidney disease. Int J Nephrol Renovasc Dis.

[4] Ogurtsova K, da Rocha Fernandes JD, Huang Y, Linnenkamp U, Guariguata L, Cho NH (2017). IDF Diabetes Atlas: Global estimates for the prevalence of diabetes for 2015 and 2040. Diabetes Res Clin Pract.

[5] Tang SCW, Yiu WH (2020). Innate immunity in diabetic kidney disease. Nature Reviews Nephrology.

[6] Saliba A, Du Y, Feng T, Garmire L (2024). Multi-Omics Integration in Nephrology: Advances, Challenges, and Future Directions. Semin Nephrol.

[7] Hu X, Chen S, Ye S, Chen W, Zhou Y (2024). New insights into the role of immunity and inflammation in diabetic kidney disease in the omics era. Front Immunol.

[8] Wang CH, Surbhi, Goraya S, Byun J, Pennathur S (2024). Fatty acids and inflammatory stimuli induce expression of long-chain acyl-CoA synthetase 1 to promote lipid remodeling in diabetic kidney disease. J Biol Chem.

[9] Milas O, Gadalean F, Vlad A, Dumitrascu V, Velciov S, Gluhovschi C (2020). Pro-inflammatory cytokines are associated with podocyte damage and proximal tubular dysfunction in the early stage of diabetic kidney disease in type 2 diabetes mellitus patients. J Diabetes Complications.

[10] Komada T, Chung H, Lau A, Platnich JM, Beck PL, Benediktsson H (2018). Macrophage Uptake of Necrotic Cell DNA Activates the AIM2 Inflammasome to Regulate a Proinflammatory Phenotype in CKD. J Am Soc Nephrol.

[11] Jia Y, Xu H, Yu Q, Tan L, Xiong Z (2021). Identification and verification of vascular cell adhesion protein 1 as an immune-related hub gene associated with the tubulointerstitial injury in diabetic kidney disease. Bioengineered.

[12] Yang WX, Liu Y, Zhang SM, Wang HF, Liu YF, Liu JL (2022). Epac activation ameliorates tubulointerstitial inflammation in diabetic nephropathy. Acta Pharmacol Sin.

[13] Park PG, Hwang J, Kim Y, Hong M, Yun D, Yoon H (2025). Inflammatory Milieu by Crosstalk between Glomerulus and Proximal Tubular Cells in Type 2 Diabetes Mellitus Kidney Disease. Diabetes Metab J.

[14] Palau V, Villanueva S, Jarrín J, Benito D, Márquez E, Rodríguez E (2021). Redefining the Role of ADAM17 in Renal Proximal Tubular Cells and Its Implications in an Obese Mouse Model of Pre-Diabetes. Int J Mol Sci.

[15] Rana R, Manoharan J, Elwakiel A, Zimmermann S, Lindquist JA, Gupta D (2024). Glomerular-tubular crosstalk via cold shock Y-box binding protein-1 in the kidney. Kidney Int.

[16] Dai CY, Tsai YM, Chang CY, Tsai HP, Wu KL, Wu YY (2024). Reconstruction of the Hepatic Microenvironment and Pathological Changes Underlying Type II Diabetes through Single-Cell RNA Sequencing. Int J Biol Sci.

[17] Tsai YC, Kuo MC, Huang JC, Chang WA, Wu LY, Huang YC (2023). Single-cell transcriptomic profiles in the pathophysiology within the microenvironment of early diabetic kidney disease. Cell Death Dis.

[18] Levey AS, Stevens LA, Schmid CH, Zhang YL, Castro AF 3rd, Feldman HI (2009). A new equation to estimate glomerular filtration rate. Ann Intern Med.

[19] Lee HJ, Choi YR, Ko JH, Ryu JS, Oh JY (2024). Defining mesenchymal stem/stromal cell-induced myeloid-derived suppressor cells using single-cell transcriptomics. Mol Ther.

[20] Adewunmi O, Shen Y, Zhang XH, Rosen JM (2023). Targeted Inhibition of lncRNA Malat1 Alters the Tumor Immune Microenvironment in Preclinical Syngeneic Mouse Models of Triple-Negative Breast Cancer. Cancer Immunol Res.

[21] Chen LX, Lu SR, Wu ZH, Zhang EX, Cai QQ, Zhang XJ (2024). Innate immunity in diabetic nephropathy: Pathogenic mechanisms and therapeutic targets. MedComm - Future Medicine.

[22] Fang Z, Zhong B, Shi Y, Zhou W, Huang M, French SW (2025). Single-cell transcriptomic analysis reveals characteristic feature of macrophage reprogramming in liver Mallory-Denk bodies pathogenesis. J Transl Med.

[23] Kim D, An L, Moon J, Maymi VI, McGurk AI, Rudd BD (2023). Ccr2+ Monocyte-Derived Macrophages Influence Trajectories of Acquired Therapy Resistance in Braf-Mutant Melanoma. Cancer Res.

[24] Fu J, Lee K, Chuang PY, Liu Z, He JC (2015). Glomerular endothelial cell injury and cross talk in diabetic kidney disease. Am J Physiol Renal Physiol.

[25] Van Mulders L, Vanden Broecke E, De Paepe E, Mortier F, Vanhaecke L, Daminet S (2025). Metabolomics reveals alterations in gut-derived uremic toxins and tryptophan metabolism in feline chronic kidney disease. Vet Q.

[26] Zhou LJ, Yang DW, Ou LN, Guo XR, Wu BL (2020). Circulating Expression Level of LncRNA Malat1 in Diabetic Kidney Disease Patients and Its Clinical Significance. J Diabetes Res.

[27] Tello-Flores VA, Valladares-Salgado A, Ramírez-Vargas MA, Cruz M, del-Moral-Hernández O, Cahua-Pablo JÁ (2020). Altered levels of MALAT1 and H19 derived from serum or serum exosomes associated with type-2 diabetes. Non-coding RNA Research.

[28] Liu JY, Yao J, Li XM, Song YC, Wang XQ, Li YJ (2014). Pathogenic role of lncRNA-MALAT1 in endothelial cell dysfunction in diabetes mellitus. Cell Death & Disease.

[29] Wang Y, Zhang C, Liu T, Yu Z, Wang K, Ying J (2024). Malat1 regulates PMN-MDSC expansion and immunosuppression through p-STAT3 ubiquitination in sepsis. Int J Biol Sci.

[30] Adewunmi O, Shen Y, Zhang XH, Rosen JM (2023). Targeted Inhibition of lncRNA Malat1 Alters the Tumor Immune Microenvironment in Preclinical Syngeneic Mouse Models of Triple-Negative Breast Cancer. Cancer Immunol Res.

[31] Garrido-Trigo A, Corraliza AM, Veny M, Dotti I, Melón-Ardanaz E, Rill A (2023). Macrophage and neutrophil heterogeneity at single-cell spatial resolution in human inflammatory bowel disease. Nature Communications.

[32] Xu R, Vujić N, Bianco V, Reinisch I, Kratky D, Krstic J (2024). Lipid-associated macrophages between aggravation and alleviation of metabolic diseases. Trends in Endocrinology & Metabolism.

[33] Guan F, Wang R, Yi Z, Luo P, Liu W, Xie Y (2025). Tissue macrophages: origin, heterogenity, biological functions, diseases and therapeutic targets. Signal Transduction and Targeted Therapy.

[34] Ganguly S, Rosenthal SB, Ishizuka K, Troutman TD, Rohm TV, Khader N (2024). Lipid-associated macrophages' promotion of fibrosis resolution during MASH regression requires TREM2. Proc Natl Acad Sci U S A.

[35] Fu J, Sun Z, Wang X, Zhang T, Yuan W, Salem F (2022). The single-cell landscape of kidney immune cells reveals transcriptional heterogeneity in early diabetic kidney disease. Kidney Int.

[36] Subramanian A, Vernon KA, Zhou Y, Marshall JL, Alimova M, Arevalo C (2024). Protective role for kidney TREM2(high) macrophages in obesity- and diabetes-induced kidney injury. Cell Rep.

[37] Awad AS, Kinsey GR, Khutsishvili K, Gao T, Bolton WK, Okusa MD (2011). Monocyte/macrophage chemokine receptor CCR2 mediates diabetic renal injury. Am J Physiol Renal Physiol.

[38] Zhu M, Zhang Z, Chen Z, Xu Y, Wu J, Che X (2022). Single-cell RNA landscape of cell fate decision of renal proximal tubular epithelial cells and immune-microenvironment in kidney fibrosis. Clin Transl Med.

[39] Li Y, Zhang L, Wu D, Zhang Z, Zhou Y, Li J (2025). Kynurenic Acid, a Small Foodborne Molecule with the Potential to Affect Human Health. J Agric Food Chem.

[40] Wang Y, Zhang Y, Wang W, Zhang Y, Dong X, Liu Y (2025). Diverse Physiological Roles of Kynurenine Pathway Metabolites: Updated Implications for Health and Disease. Metabolites.

[41] Zhao H, Huang X, Liu Z, Pu W, Lv Z, He L (2021). Pre-existing beta cells but not progenitors contribute to new beta cells in the adult pancreas. Nat Metab.

[42] Yang S, Gong W, Wang Y, Hao C, Guan Y (2024). Unraveling the nexus of NAD+ metabolism and diabetic kidney disease: insights from murine models and human data. Front Endocrinol (Lausanne).

[43] Saliba A, Debnath S, Tamayo I, Lee HJ, Ragi N, Das F (2025). Quinolinic acid potentially links kidney injury to brain toxicity. JCI Insight.

[44] Klawitter J, Jackson MJ, Smith PH, Hopp K, Chonchol M, Gitomer BY (2023). Kynurenines in polycystic kidney disease. J Nephrol.

[45] Wu IW, Tsai TH, Lo CJ, Chou YJ, Yeh CH, Cheng ML (2022). Discovery of a Biomarker Signature That Reveals a Molecular Mechanism Underlying Diabetic Kidney Disease via Organ Cross Talk. Diabetes Care.

[46] Della Chiesa M, Carlomagno S, Frumento G, Balsamo M, Cantoni C, Conte R (2006). The tryptophan catabolite L-kynurenine inhibits the surface expression of NKp46- and NKG2D-activating receptors and regulates NK-cell function. Blood.

[47] Gadupudi GS, Chung KT (2011). Comparative genotoxicity of 3-hydroxyanthranilic acid and anthranilic acid in the presence of a metal cofactor Cu (II) in vitro. Mutat Res.

