# Supplementary material for: Immune Cell Infiltration and Kynurenine Pathway Activation Define Early Injury and Progression in Diabetic Nephropathy
Source: Int J Biol Sci. 2026 Jan 8;22(3):1389–406. doi: 10.7150/ijbs.122164 (PMC12837742; doi:10.7150/ijbs.122164)
Supplement: Supplementary file 1 — Supplementary figures and tables. [file ijbsv22p1389s1.pdf]

Supplementary Figure 1. Additional characterization of MDSC subsets and validation of molecular markers.

A The heatmap of MDSC subsets markers.

B Violin plots showing the expression of LCN2, SRGN, S100A8, and S100A9 across $MKI67^{+}LY6C2^{+}$ and $CCL4^{+}$ MDSC subsets, indicating shared expression of key effector molecules involved in immunosuppressive function.

C Expression profile of MALAT1 across identified MDSC subsets from single-cell RNA-seq data, demonstrating consistently high expression in both proliferative and differentiated populations.

Supplementary Figure 2. The dynamics of circulating inflammatory mediators during diabetic nephropathy progression.

A-E Serum levels of **(A)** CXCL2, **(B)** S100A9, **(C)** SRGN, **(D)** LCN2 and **(E)** IL-1β in diabetic mice at early, mid, and late stages of diabetic nephropathy (DN). CXCL2 and S100A9 increased during the early stage, SRGN was elevated in the mid-stage, and LCN2 showed a marked rise in the late stage of DN. Data represent mean ± SE; P values were calculated using one-way ANOVA with post hoc test. *, $p < 0.05$, **, $p < 0.01$, ***, $p < 0.001$, ns, no significant. Expression values are presented as mean ± SD. In vitro experiments were performed with at least three independent replicates.

Supplementary Table 1. Target sequence of materials utilized in the study

Supplementary Table 2. The gene list of MDSC score

Supplementary Table 3. The clinical characteristics of study participates

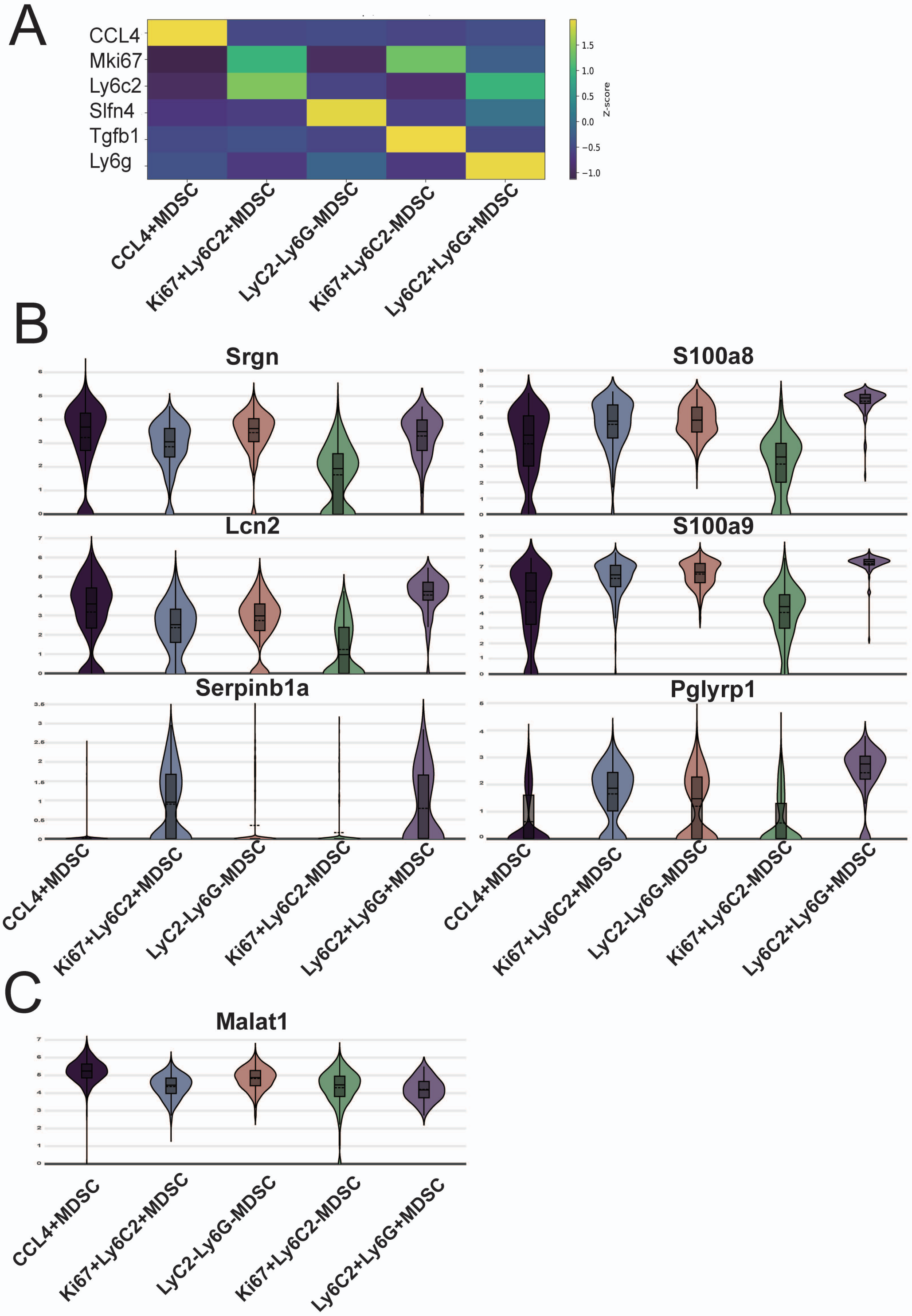
A
CCL4
Mki67
Ly6c2
Slfn4
Tgfb1
Ly6g
Z-score
CCL4+MDSC
Ki67+Ly6C2+MDSC
LyC2-Ly6G-MDSC
Ki67+Ly6C2-MDSC
Ly6C2+Ly6G+MDSC
B
Srgn
S100a8
Lcn2
S100a9
Serpinb1a
Pglyrp1
C
Malat1

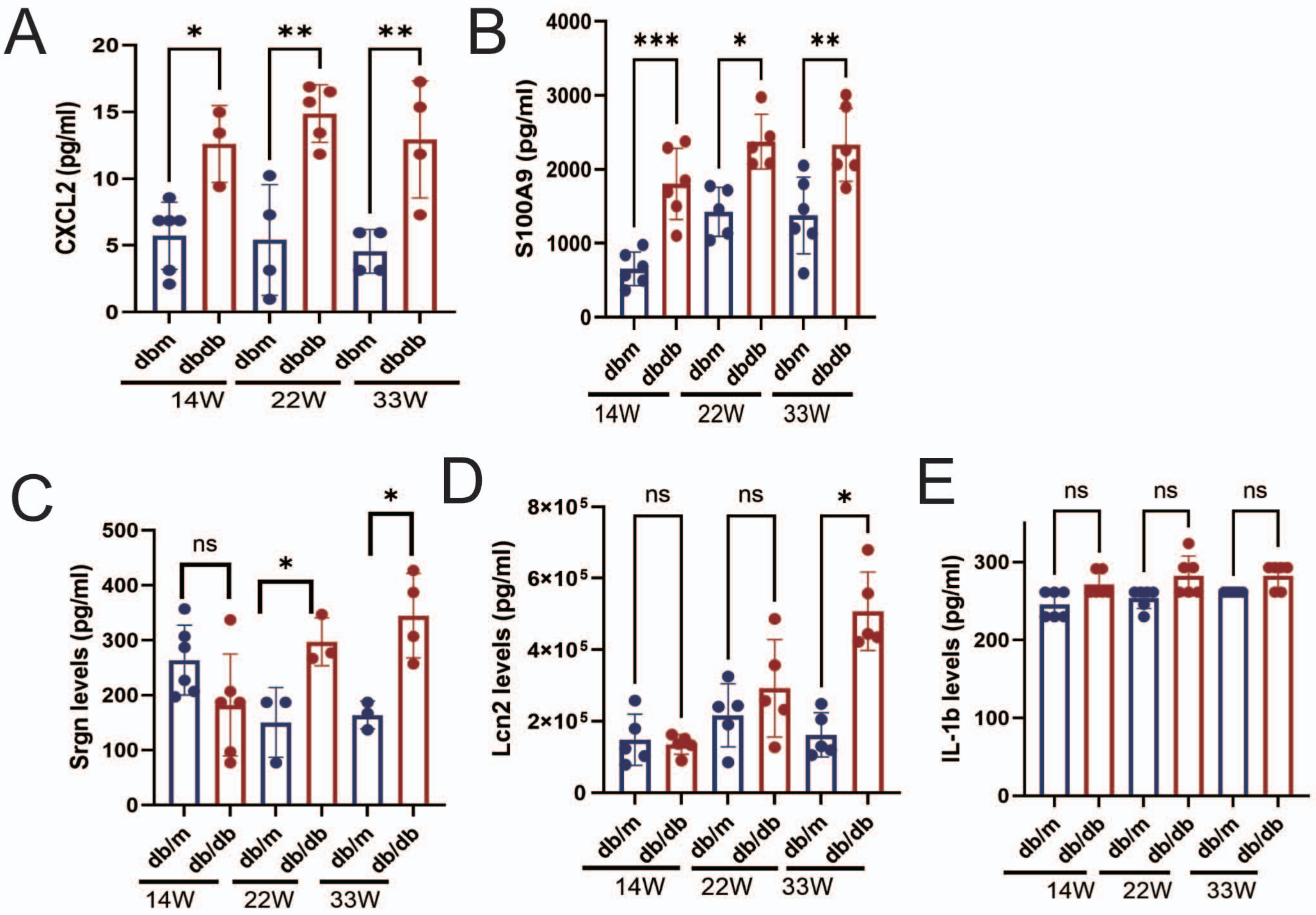
A
CXCL2 (pg/ml)
dbm
dbdb
14W
22W
33W
B
S100A9 (pg/ml)
C
Srgn levels (pg/ml)
db/m
db/db
D
Lcn2 levels (pg/ml)
E
IL-1b levels (pg/ml)
ns

Table S1. Target sequence of materials utilized in the study

Gene	Primers
18srRNA_Mus	Forward,<br>5’- CGGCTACCACATCCAAGGAA-3’<br>Reverse,<br>5’- GCTGGAATTACCGCGGCT-3’
MALAT1_Mus	Forward,<br>5’- AGCAGGCATTGTGGAGAGGA -3’<br>Reverse,<br>5’- ATGTTGCCGACCTCAAGGAA-3’
TREM2_Mus	Forward,<br>5’- CGGAATGGGAGCACAGTCAT-3’<br>Reverse,<br>5’- TCTAGAGGGTCCTCCAGCAC-3’
CD9_Mus	Forward,<br>5’- ATATGGCGTTGGACTGCTGT -3’<br>Reverse,<br>5’- AAGATCATCACCACGGCGAT-3’
ARG1_Mus	Forward,<br>5’- CAACACTCCCCTGACAACCA-3’<br>Reverse,<br>5’- CTCGCAAGCCAATGTACACG -3’
Lcn2_Mus	Forward,<br>5’- ACTGAATGGGTGGTGAGTGT-3’<br>5’-tgtcacctccatcctggtca-3’<br>Reverse,<br>5’- GGGAGTGCTGGCCAAATAAG -3’<br>5’-TGGCGAACTGGTTGTAGTCC-3’
S100A9_Mus	Forward,<br>5’- ACTGGGCTTACACTGCTCTT-3’<br>5’-gtggaagcacagttggcaac-3’<br>Reverse,<br>5’- TTCCTTCTTGCTCAGGGTGT -3’<br>5’-TGGGTTGTTCTCATGCAGCT-3’
NC siRNA_Mus	Target Sequences<br>UGGUUUACAUGUCGACUAA,<br>UGGUUUACAUGUUGUGUGA,<br>UGGUUUACAUGUUUUCUGA,

	UGGUUUACAUGUUUUCCUA
Malat1<br>siRNA_Mus	Target Sequences<br>GUUUAAAUGCUUACGAUCA<br>AUAGAGUAGCUUAUCGAAA<br>GGUUAGAGAAGGCGUGUAC<br>GAGAGCAUGCGGUGCGGUA

Table S2. The gene list of MDSC

Cxcl3
Ccl4
Basp1
Il1rn
Il1b
Ccrl2
Ccl3
Camp
Gadd45b
Saa3
Ngp
Hcar2
Ifrd1
Ltf
Cd177
AA467197
Adpgk
Anxa1
Dstn
Nfkbia
Lcn2
Btg1
S100a8
S100a9
Arhgdib
Mmp8
Jun
Lyz2

Table S3. The clinical characteristics of study participates

	Entire Cohort (n=497)	eGFR ≥ 60 ml/min/1.73m^2 (n=387)	eGFR < 60 ml/min/1.73m^2 (n=110)	P-value
**Clinical characteristics**				
Age, year	60.7±12.2	58.7±11.5	68.0±11.6	<0.001
Sex (male), %	53.5	50.9	63.6	0.02
Smoke, %	22.9	21.8	27.3	0.22
Alcohol, %	18.9	18.4	21.8	0.42
Hypertension, %	57.1	51.4	76.4	<0.001
Gout, %	7.7	5.7	12.7	0.01
Hyperlipidemia, %	69.3	67.7	72.7	0.31
T2D duration, year	9.3±8.5	8.1±7.3	13.1±10.6	<0.001
Body mass index, kg/m^2	26.6±4.5	26.8±4.5	26.0±4.3	0.10
**Medication**				
Sulfonylurea, %	43.5	39.5	58.2	0.001
DPP4 inhibitor, %	62.4	57.1	80.9	<0.001
Glucophage, %	79.3	81.9	73.6	0.06
SGLT2 inhibitor, %	6.3	5.9	4.5	0.57
Actos, %	6.1	5.7	7.3	0.53
Insulin, %	19.7	16.5	31.8	<0.001
Statin, %	57.9	55.0	67.3	0.02
Calcium channel blocker, %	22.6	16.8	40.0	<0.001
Beta blocker, %	21.7	16.3	40.0	<0.001
ACEI/ARB, %	45.3	40.3	62.7	<0.001
**Kynurenine pathway**				
Tryptophan, µM	55.40(48.00,63.03)	56.78(49.93,64.12)	50.38(43.14,56.70)	<0.001
Kynurenine, µM	1.32(1.11,1.62)	1.24(1.04,1.49)	1.71(1.46,2.05)	<0.001
Kynurenic acid, µM	0.06(0.04,0.08)	0.05(0.04,0.07)	0.11(0.06,0.14)	<0.001
3-Hydroxykynurenine, µM	0.04(0.03,0.06)	0.04(0.03,0.05)	0.06(0.05,0.08)	<0.001
Quinolinic acid, µM	0.43(0.33,0.55)	0.39(0.31,0.51)	0.55(0.44,0.75)	<0.001
Xanthurenic acid, µM	0.30(0.25,0.34)	0.39(0.31,0.51)	0.27(0.23,0.31)	<0.001
**Laboratory parameters**				
Albumin, g/dl	4.5±0.3	4.5±0.3	4.4±0.4	<0.001
Hemoglobin, g/dl	13.7±1.8	13.9±1.6	12.8±1.9	<0.001
Uric acid, mg/dl	5.9±1.8	5.7±1.4	13.1±10.6	0.001
GOT, IU/dl	30.3±14.7	31.0±15.2	28.5±13.2	0.10

GPT, IU/dl	33.6±24.0	35.4±25.2	28.4±19.0	0.002
Blood urea nitrogen	14.6(11.5,18.7)	13.4(10.9,16.3)	22.5(17.9,27.5)	<0.001
Creatinine, mg/dl	0.9±0.4	0.8±0.2	1.4±0.3	<0.001
eGFR, ml/1.73m^2/yr	81.2±26.6	94.8±14.3	49.9±9.7	<0.001
Cholesterol, mg/dl	170.6±40.2	172.4±36.4	164.4±49.4	0.011
Triglyceride, mg/dl	118(88,179)	116(85,179)	124(91,173)	0.42
HbA1c, %	7.0(6.5,7.9)	7.0(6.5,7.9)	7.0(6.4,7.7)	0.75
Urinary ACR, mg/g	16.6(6.7,62.6)	12.5(5.6,36.8)	55.3(10.9,362.7)	<0.001

Data are expressed as numbers (percentages) for categorical variables and means ± SDs or medians (25th, 75th percentiles) for continuous variables as appropriate.

Abbreviations: ACEI, angiotensin converting enzyme inhibitors; ACR, albumin-creatinine ratio; ARB, angiotensin II receptor blockers; DM, diabetes mellitus; DPP-4, dipeptidyl peptidase 4; eGFR, estimated glomerular filtration rate; GOT, glutamate oxaloactate transminase; GPT, glutamate pyruvate transaminase; HbA1c, glycated hemoglobin; SGLT2, sodium–glucose cotransporter 2; T2D, type 2 diabetes mellitus
